# Transdiagnostic Approaches to Mental Health Problems: Current Status and Future Directions

**DOI:** 10.1037/ccp0000482

**Published:** 2020-03

**Authors:** Tim Dalgleish, Melissa Black, David Johnston, Anna Bevan

**Affiliations:** 1 Medical Research Council Cognition and Brain Sciences Unit, University of Cambridge, and The Cambridgeshire and Peterborough NHS Foundation Trust, Cambridge, United Kingdom

**Keywords:** transdiagnostic, mental health, classification, biopsychosocial processes, clinical interventions

## Abstract

Despite a longstanding and widespread influence of the diagnostic approach to mental ill health, there is an emerging and growing consensus that such psychiatric nosologies may no longer be fit for purpose in research and clinical practice. In their place, there is gathering support for a “transdiagnostic” approach that cuts across traditional diagnostic boundaries or, more radically, sets them aside altogether, to provide novel insights into how we might understand mental health difficulties. Removing the distinctions between proposed psychiatric taxa at the level of classification opens up new ways of classifying mental health problems, suggests alternative conceptualizations of the processes implicated in mental health, and provides a platform for novel ways of thinking about onset, maintenance, and clinical treatment and recovery from experiences of disabling mental distress. In this Introduction to a Special Section on *Transdiagnostic Approaches to Psychopathology*, we provide a narrative review of the transdiagnostic literature in order to situate the Special Section articles in context. We begin with a brief history of the diagnostic approach and outline several challenges it currently faces that arguably limit its applicability in current mental health science and practice. We then review several recent transdiagnostic approaches to classification, biopsychosocial processes, and clinical interventions, highlighting promising novel developments. Finally, we present some key challenges facing transdiagnostic science and make suggestions for a way forward.

## What is the public health significance of this article?

Traditional diagnostic systems may no longer be fit for purpose for classifying mental ill health, facilitating understanding of its core underlying biopsychosocial processes, nor driving clinical developments. Here we propose that ‘transdiagnostic’ approaches have the potential to better represent the clinical and scientific reality of mental health problems, reflecting the complexity, dimensionality and comorbidity that is the norm in clinical practice.

Distress and suffering are an existential cornerstone of the human condition, yet how we reflect upon and describe the extremes of our mental duress has varied enormously across history. For more than 100 years, certainly in the West, the predominant means of conceptualizing mental health struggles has been to categorize them within formal taxonomic systems (Kendler, 2009), organized according to hypothetical distinctions between different sets of signs and symptoms, and compiled into comprehensive compendia of psychiatric diagnoses. The current leading such taxonomies—the *Diagnostic and Statistical Manual of Mental Disorders* (*DSM*; now in its 5th edition) and the International Classification of Diseases (ICD; now in its 11th edition)—are long-established, have global reach, and exert a profound influence over the ways in which we understand, assess, and manage mental ill health.

Despite the historical momentum and widespread influence of the diagnostic rubric, there is an emerging and growing consensus that such psychiatric nosologies may be reaching the limits of their research and clinical utility. In their place, there is gathering support for a “transdiagnostic” approach that cuts across the traditional diagnostic boundaries or, more radically, sets them aside altogether, to provide novel insights into how we might understand mental health difficulties. This transdiagnostic approach extends beyond issues of taxonomy. Removing the distinctions between proposed psychiatric taxa at the level of classification opens up new ways of conceptualizing the underlying theories and processes implicated in mental ill health and provides a platform for novel ways of thinking about onset, maintenance, and clinical treatment and recovery from experiences of disabling mental distress.

The transdiagnostic field is nascent yet fast-developing. This Special Section of the *Journal of Consulting and Clinical Psychology* provides a timely opportunity to take stock of the current state of transdiagnostic science. In this introduction we review where the field has come from and where it is heading, highlighting issues that require some resolution and offering recommendations for progress.

## Diagnostic and Transdiagnostic Approaches

Although attempts to classify mental health difficulties date back several thousand years, formalized diagnostic models only emerged properly from the biological and Linnaen botanical classification systems of the 19th century. Most prominently, Kraepelin’s *Compendium der Psychiatrie* in 1883 (Compton & Guze, 1995) exerted a profound influence on the development of the emerging field of clinical psychiatry especially in the United States, laying the foundation for the publication of the first edition of the *DSM* in 1952 (*DSM-I*, American Psychiatric Association, 1952). In 1980, the publication of the *DSM–III* outlined for the first time a thorough multiaxial diagnostic system with carefully operationalized criteria for a wide range of disorders, with no allegiance to any theoretical approach aside from a broad biomedical model. The *DSM–III* was hailed as a “paradigm shift” (Blashfield, Keeley, Flanagan, & Miles, 2014) for diagnostic psychiatry, rescuing the profession “. . . from unreliability and the oblivion of irrelevancy” (Frances, 2009, p. 2). The current instantiation of the *DSM*—the *DSM–5* – appeared in 2013 after a 14 year gestation and runs to 947 pages covering some 541 diagnostic categories (up from 106 in the *DSM-I*; American Psychiatric Association, 2013).

The *DSM* and ICD have evolved into self-perpetuating systems that now govern and define all aspects of how we conceptualize mental health. They provide an organizing framework for virtually all core texts in psychiatry, clinical psychology, and abnormal psychology (Cosgrove, Krimsky, Vijayaraghavan, & Schneider, 2006; Marecek & Hare-Mustin, 2009), they guide mental health training across the helping professions, and they define how we assess, manage and treat mental health problems worldwide. The diagnostic systems that they enshrine have created a form of “epistemic prison” (Hyman, 2010) that constrains health insurance and pharmaceutical industry practices, is sanctioned and supported by government and legal policies, and dominates social and public discourse about mental health and illness, as reflected in art, literature and the visual media (Ussher, 2010).

There are many factors underscoring this rise to dominance. Some are certainly sociopolitical (Kawa & Giordano, 2012; Khoury, Langer, & Pagnini, 2014) with diagnoses offering a biomedical legitimacy to discourse about mental ill health that has a broad academic, professional, and social appeal. Others are more pragmatic as, without doubt, the diagnostic paradigm offers some clear benefits to clinical and research practice: It provides a *lingua franca* for describing clusters of symptoms that facilitates communication between users of services, clinicians and researchers; it sets out a common metric for research programs; and it provides an organizing principle for the development and evaluation of diagnosis-led assessment and treatment approaches (Hayes & Hofmann, 2018). Finally, for some, the biomedical model at the heart of the diagnostic approach also brings a legitimacy to the suffering that is experienced, reducing stigma and deflecting pejorative judgments that mental ill health reflects some form of personal weakness on the part of the diagnosed.

Despite these advantages of the diagnostic paradigm, there is a gathering apprehension that the taxonomic approach instantiated in the *DSM* and ICD runs counter to the available clinical and research evidence and may hamper our understanding of mental ill health and consequently how we manage and treat mental distress (Insel, 2014; Kotov et al., 2017). Here we touch briefly on seven areas of concern that have currency within this debate. We focus predominantly on so-called common mental health problems (Craig & Boardman, 1997), captured by the various diagnoses of mood disorder, anxiety disorder, stressor-related disorders, and obsessive–compulsive disorders within the diagnostic manuals, but the arguments of course extend beyond these presentations.

## Seven Challenges for the Diagnostic Paradigm

### The Underlying Biopsychosocial Processes are Transdiagnostic

A perhaps unintended consequence of the psychiatric diagnostic paradigm is the notion that diagnoses somehow capture or reflect the underlying reality of the world, carving nature at its joints and identifying natural kinds of “mental disorder.” This idea stems from general medicine, where the majority of physical illnesses and diseases reflect qualitatively different states of health with one or a small number of identifiable and discrete causes. This is not the case for mental health problems where it is generally accepted that causes are not only complex, multiple and interactive but as yet poorly understood (Kendler, 2008, 2012). What we know is that mental ill health prototypically emerges from an interplay between myriad biological, behavioral, psychosocial, and cultural processes that do not respect established diagnostic boundaries, where the interactions are multifarious, and modulated by an individual’s lifelong experiences of the world.

### The Symptom Space is Dimensional

Within the diagnostic manuals, symptoms are thresh-holded, imposing binary notions of “present” versus “absent” (Regier, Kuhl, & Kupfer, 2013). Groupings of symptoms deemed to be present then comprise the different diagnoses albeit with guidance on severity qualifiers for “mild,” “moderate,” and “severe” manifestations of individual diagnoses. However, evidence overwhelmingly suggests that mental health symptoms are not all-or-none phenomena, but are better conceptualized along continuous dimensions within the population as opposed to these distinct categorical entities (Brown, Campbell, Lehman, Grisham, & Mancill, 2001; Kessler, Chiu, Demler, Merikangas, & Walters, 2005). Indeed, there is a lack of compelling evidence for even a single symptom or disorder being a distinct category (Haslam, Holland, & Kuppens, 2012).^1^

This imposition of artificial categories onto a multidimensional space inevitably sacrifices much of the richness of the available clinical information, contributing to diagnostic instability with symptoms falling above or below imposed thresholds over time, as well as reduced interrater reliability as assessors struggle to elucidate whether marginal symptoms cross the designated thresholds (Markon, Chmielewski, & Miller, 2011). Most importantly, many individuals experiencing psychological distress fall short of the criteria for any diagnosis, despite a manifest need for care (Kotov et al., 2018).

### Rampant Comorbidity and Poor Discrimination Between Supposedly Different Disorders

Comorbidity—when someone presents with a profile of problems that satisfy the criteria for more than one diagnosis at a time—is not a problem per se for the diagnostic model, with the notion of secondary diagnoses and/or complications of primary problems woven into the fabric of psychiatric taxonomies since the outset. However, of greater concern is that epidemiological findings reveal that comorbidity among psychiatric diagnoses is the rule rather than the exception, and single, uncomplicated clinical presentations are actually relatively scarce (Kessler et al., 2005). Such comorbidity is associated with greater clinical severity and functional impairment (Wittchen et al., 2011), higher rates of symptom chronicity (Rapaport, Clary, Fayyad, & Endicott, 2005) and a greater detriment to overall quality of life (Hofmeijer-Sevink et al., 2012). This “rampant” (Clark, Cuthbert, Lewis-Fernández, Narrow, & Reed, 2017) diagnostic comorbidity suggests that the normative coexistence of psychiatric disorders must, to a considerable extent, be an artifact (e.g., Maj, 2005) arising from the structure of the categorical classification system itself, rather than the co-occurrence of genuinely separable syndromes (van Loo & Romeijn, 2015).

### Massive Heterogeneity Within Diagnoses

Formal diagnoses of different disorders typically comprise a number of criteria—clusters of conceptually similar symptoms that are heuristically grouped together. Most criteria contain more than one symptom and one or more of these symptoms would need to be present for the criterion to be met. Typically, the overall diagnosis then further depends on a specified number of criteria being satisfied. Even for diagnoses where criteria are not explicitly offered, diagnoses normatively require only a subset of symptoms from a larger set to be present. This polythetic checklist approach means that individuals receiving the same diagnosis can present with very different symptoms such that each diagnostic category incorporates built in heterogeneity.

To illustrate, within the *DSM–5* major depressive disorder (MDD) diagnosis, two individuals meeting criteria for MDD could potentially have only one symptom in common from the nine listed in the Manual. Indeed, when we account for all of the subsymptoms and directional qualifiers, there are 16,400 different symptom profiles that all qualify as MDD (Fried & Nesse, 2015). Such heterogeneity is orders of magnitude greater for complex criteria-based diagnoses such as posttraumatic stress disorder where there are 636,120 permutations that qualify for the diagnosis (Galatzer-Levy & Bryant, 2013).

How does this play out in actual epidemiological data? In the Sequenced Treatment Alternatives to Relieve Depression (STARnD) data (*N* = 3,703), Fried and Nesse (2015) identified 1,030 unique MDD symptom profiles, of which 864 (83.9%) were endorsed by fewer than six participants, with almost half of the profiles (501; 48.6%) endorsed by only a single individual. Indeed, the most common profile was only met by 67 people.

A primary function of any diagnostic system should be to facilitate our understanding of a complex problem space by organizing central, recurrent patterns into discrete categories. The data on heterogeneity cast doubt on whether this pragmatic aim of the psychiatric paradigm has even come close to being realized.

### Incomplete Symptom Capture

A central question for the compilers of diagnostic compendia is which symptoms to include as prototypical to delineate a given disorder given that many symptoms of mental health problems also characterize everyday life; for example, tiredness, low mood, and so on and many disorders are associated with a multiplicity of signs and symptoms. The case of depression again provides a revealing illustration. If we look at established measurement tools for depression, there are some 280 different instruments developed in the last century, of which many are still in use (Santor, Gregus, & Welch, 2006). These assessment instruments differ markedly in the signs and symptoms that they capture. For instance, Fried (2017) notes that across the seven most commonly used depression assessment tools, 52 distinct depression symptoms are measured (compared with the nine symptoms listed in the *DSM–5*), with 40% of those symptoms appearing in just one of the seven scales and only 12% appearing across all seven. Notwithstanding the fact that some of these 280 instruments may have weak clinical validity, the wide scope of clinical signs and symptoms covered suggests that there is no single set of cardinal symptoms that defines depression (and by extension other diagnostic categories) and consequently that the profile outlined in the diagnostic manuals may be overly narrow or rigid, failing to reasonably capture the range within the clinical data.^2^

### Phenotypic Plasticity Across Development and The Life Course

Mental health problems can morph across development and the life course such that individuals satisfy criteria either for different diagnoses or present differentially within the same diagnosis across time; for example, shifting between anxiety and unipolar depressive disorders (Fichter, Quadflieg, Fischer, & Kohlboeck, 2010), within anxiety disorders (Wittchen, Carter, Pfister, Montgomery, & Kessler, 2000, 2008) or within depressive disorders (Oquendo et al., 2004). Some of this phenotypic plasticity is a function of development, but the nature of the diagnostic approach itself arguably also contributes with its reliance on cross-sectional “snapshot” dichotomizations of what are in fact dimensional and dynamic symptom constructs that will wax and wane across time (Bystritsky, Nierenberg, Feusner, & Rabinovich, 2012).

### Diagnosis-Driven Clinical Intervention

A much-vaunted advantage of diagnostic taxonomies is facilitated clinical assessment, management and intervention. This has led, within the domain of psychological interventions which is our primary focus here, to the establishment of an evidence-base for a plethora of single-disorder-focused treatment approaches. These are then endorsed by diagnostically organized guidelines such as those compiled by the United Kingdom’s National Institute for Health and Care Excellence (NICE; Pilling, Whittington, Taylor, & Kendrick, 2011). Comorbid conditions are generally either glossed over, or minimally treated within these intervention packages and there little attention is paid to symptoms that fall outside of the diagnostic rubric. However, the majority of mental health treatments of all types actually appear to be effective across broad ranges of clinical populations, for example drugs such as selective serotonin reuptake inhibitors (SSRIs) and benzodiazepines, and psychological protocols such as cognitive-behavior therapy (CBT), or extinction-based approaches for anxiety-related difficulties. There is thus a mismatch between the clinical reality on the ground and the nature and scope of the recommended interventions. As a result, much of real-world clinical practice eschews the diagnosis-led treatment evidence base, preferring instead eclectic combinations of treatment elements tailored to the presentation and formulation of individual clients. This pragmatic approach enables goodness-of-fit matching of interventions to specific vulnerabilities and processes relevant to the individual, and provides a flexible treatment model that can be applied across a range of presentations including, critically, complex formulations, comorbidity, and subsyndromal or prodromal symptoms.

## A Transdiagnostic Alternative

These diverse concerns about the diagnostic approach stem somewhat independently from research and scholarship across the three intellectual domains of classification and nosology, basic biopsychosocial research, and clinical science. Perhaps unsurprisingly, therefore, the alternative transdiagnostic approaches that abnegate the traditional psychiatric paradigm have also evolved and matured somewhat separately in each of these spheres, as we highlight below.

Within each of the three domains, the degree to which the diagnostic model is forsaken and consequently the strength of the transdiagnostic proposals vary. What we shall call here “soft” transdiagnostic approaches preserve the underlying diagnostic classification while seeking to elucidate processes or develop interventions that have relevance to one or more of the diagnoses as traditionally formulated. In contrast, more radical, “hard” transdiagnostic approaches dispense with the diagnostic system altogether, seeking to replace it with alternative frames of reference that characterize mental ill health in new ways.

## Transdiagnostic Approaches to Classifying Mental Health Problems

The established diagnostic taxonomy has been generated through consensual decision-making by groups of experts under the auspices of learned bodies (Blashfield, 1984). Although this “authoritative” approach (Krueger et al., 2018) relies on some empirical data (e.g., the *DSM* field trials; Regier, Narrow, et al., 2013), this is secondary to the influence of expertise, tradition and politics. An alternative approach to the *ex cathedra* diagnostic manuals is fully empirical, focusing on the quantitative structure of signs, symptoms, and behaviors associated with mental health and distress, and deriving classification frameworks based on the resultant data.

The overwhelming weight of evidence from decades of such data-driven efforts indicates that mental health problems are best conceptualized along a series of continua rather than as discrete categories (Haslam et al., 2012; Waszczuk et al., 2017). Alternative categorical delineations of the mental health symptom space, and hybrid models that combine dimensional and discrete components, do have their proponents (Blashfield, 1984; Goekoop & Goekoop, 2014; Masyn, Henderson, & Greenbaum, 2018) but the bulk of the evidence favors dimensionality. The broadest empirical support is that these dimensions are organized hierarchically. Here, as an illustration of this approach, we focus on one attempt to generate such a hierarchical framework—the Hierarchical Taxonomy of Psychopathology (HiToP; Kotov et al., 2017).

**Figure 1 fig1:**
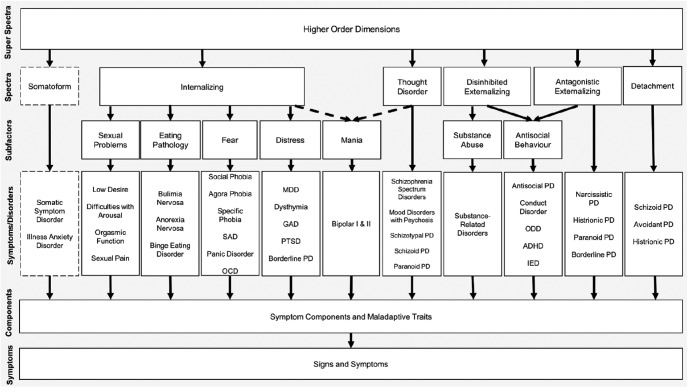
The hierarchical taxonomy of psychopathology (HiToP; Kotov et al., 2017). MDD = major depressive disorder; PD = personality disorder.

The HiToP architecture (see Figure 1) emerges from multiple data sets with a combined sample size of over 100,000. HiToP includes a general factor of psychopathology at the apex, followed by a number of broad dimensions of disorder at the next level—spectra—including, for example, internalizing and disinhibited/externalizing problems. These higher-level spectra sit above lower-order dimensions that align with subsets of traditional diagnoses or disorders that tend to co-occur (Slade & Watson, 2006). These syndrome-level dimensions themselves sit above lower-order levels of symptom components and individual signs and symptoms.[Fig-anchor fig1]

Alternative approaches other than HiToP favor a bifactor structure where lower-order dimensions are not nested within broader higher-order factors, but instead explain unique residual variance that is not accounted for by the higher-order constructs (e.g., Caspi et al., 2014; Lahey et al., 2012; Snyder, Young, & Hankin, 2017). The breadth of ambition varies across dimensional approaches, with HiToP aspiring eventually to guide clinical assessment and intervention (Ruggero et al., 2019) and accommodate dimensions of personality as well as developmental trajectories, including cognitive risk dimensions (Kotov et al., 2017; Schweizer, Snyder, Young, & Hankin, 2020), whereas other approaches are somewhat narrower in scope (Craddock & Owen, 2010).

There are myriad advantages to such dimensional frameworks. They are firmly grounded in empirical science, therefore not only reflecting the underlying dimensional nature of the explicanda but also sidestepping many of the sociopolitical concerns that dog the traditional “top-down” diagnostic model. They appear to have greater reliability than discrete diagnoses, both between raters (Markon et al., 2011) and across time (e.g., Eaton et al., 2013). A dimensional structure also seems to map more closely onto underlying biopsychosocial processes (e.g., Weinberg, Kotov, & Proudfit, 2015) and both genetic (e.g., Hicks, Foster, Iacono, & McGue, 2013; Kendler et al., 2011) and environmental (e.g., Keyes et al., 2012) vulnerability factors. Dimensions also provide a more compelling framework for thinking about the course of mental health difficulties as they fluctuate across time, in contrast to categorical thresholds with their imprecise notions of “in episode,” remission, and recovery (Kotov et al., 2017).

The dimensional architecture is additionally able to deal with many of the structural issues that bedevil diagnostic taxonomies. Comorbidity is accounted for by higher-order dimensions that code regularities across related lower-order constructs and the dimensional nature of lower-order component and symptom-level layers provides a level of description that captures the broad heterogeneity that characterizes diagnoses.

Clinically, the dimensional taxonomy has potential advantages. Arguably, dimensional approaches lie at the heart of clinical practice where critical triage decisions revolve around whether to intervene and, if so, what level of intervention is indicated, based on severity of symptoms or clusters of symptoms (Helzer, Kraemer, & Krueger, 2006; Ruggero et al., 2019; Waszczuk et al., 2017).

A final potential strength of the dimensional approach, as operationalized within HiToP, is that it reflects the efforts of a consortium of like-minded researchers, numbering more than 70, with backgrounds in diverse disciplines. The size of the task—to comprehensively map the symptom space of mental health—requires this kind of collaborative effort. The historical and political momentum behind the currently dominant diagnostic model requires a broad, robust, and consensus set of counterproposals of the sort that are unlikely to emerge from more localized classification efforts.

The dimensional hierarchical paradigm is not without its issues. At present, despite their overt reliance on data-driven, empirical analysis, the focus of dimensional approaches is nevertheless predominantly to reorganize the same symptom space that is the purview of the diagnostic approach. Consequently, the explicanda remain a function of the nonempirical top-down choices about which symptoms are central to mental ill health, that originate with and characterize the psychiatric model. Potentially, empirically driven or theoretically aligned approaches to identifying the range of relevant signs and symptoms would generate a somewhat different hierarchical structure.

A related issue concerns the interaction between different dimensional components. Clinical data reveal that symptoms interact with each other as networks of associated problems in theoretically meaningful ways; for example, sleep problems might drive low mood and poor concentration (Borsboom, 2017; Borsboom & Cramer, 2013). Indeed, proponents of network approaches argue that apparent higher-order dimensions or categories of symptoms such as diagnoses may simply emerge from the proliferation of chain reactions of symptoms reciprocally activating each other (Cramer, Waldorp, van der Maas, & Borsboom, 2010). According to this analysis, the nature and dynamics of these interactive networks should be the prime clinical focus (McNally, 2016). At present, hierarchical taxonomical approaches are mostly agnostic about how within- and across-levels of such taxonomies the different components dynamically interact across time, although in principle the two approaches are not mutually exclusive and a recent network analysis has attempted to integrate the two (Funkhouser et al., in press).

It is also, by definition, unclear what constitutes clinical “caseness” within a dimensional approach. This is not insurmountable—clinical cut-offs can easily be projected onto individual dimensions—but the issue quickly becomes challenging once multidimensional constructs are considered together.

Finally, although the reliance on data-driven approaches to compile the hierarchical taxonomy represents an advance over the decision-by-committee model of the diagnostic manuals, it nevertheless remains atheoretical. Without theory, there is an explanatory vacuum concerning why dimensions are organized as they are, or about what determines an individual’s clinical presentation in terms of his or her positioning on multiple dimensions. In terms of psychological interventions, the absence of theory makes it difficult to see how the dimensional model can easily translate into novel clinical approaches. Psychological interventions are not conceptualized in terms of symptoms or syndromes, but rather to act on identified precipitating and maintaining processes that are deemed to be mutable. Without adequate theory driving intervention development, dimensional approaches will likely struggle in the same way as diagnostic approaches to bridge the translational gap (though see Ruggero et al., 2019, for a recent discussion).

## Transdiagnostic Biopsychosocial Processes

As already noted, a foundation stone of the transdiagnostic approach is that the risk, protective, and maintenance factors and processes implicated in mental health problems, whether they be biological, socioenvironmental, or psychological, show no specificity for particular diagnostic disorders but rather appear to operate across traditional nosological boundaries (Buckholtz & Meyer-Lindenberg, 2012). Quantitative and molecular genetic studies (e.g., Otowa et al., 2016; Pettersson, Larsson, & Lichtenstein, 2016), structural and functional brain research (e.g., Baker et al., 2019; Goodkind et al., 2015; McTeague et al., 2017; Meyer, 2017; Shanmugan et al., 2016; Swartz, Knodt, Radtke, & Hariri, 2015), studies investigating the influence of environmental factors such as poverty, discrimination, loneliness, aversive parenting, and childhood trauma or maltreatment (Green et al., 2010; Käll et al., 2020; Keyes et al., 2012; Lehavot & Simoni, 2011; Sunderland et al., 2016; Wiggins, Mitchell, Hyde, & Monk, 2015), and investigations of psychological processes (Gellatly & Beck, 2016; Hayes & Hofmann, 2018; Shanmugan et al., 2016; Sharma et al., 2017; Wahl et al., 2019), all support biopsychosocial factors that transcend diagnostic precincts. Indeed, to date no biological markers or cognitive processes have been identified that are uniquely associated with a specific disorder (Kupfer, First, & Regier, 2002; Widiger & Samuel, 2005).

This transdiagnostic picture led the National Institute of Mental Health (NIMH) in 2009 to “*Develop, for research purposes, new ways of classifying mental disorders based on dimensions of observable behavior and neurobiological measures*” in the form of the Research Domain Criteria (RDoC), thus abandoning the diagnostic approach that it had championed for the previous three decades. The RDoC framework (Insel et al., 2010) is an epistemic infrastructure that parses mental health complexity into six supraordinate domains (see Supplementary Table S1): positive valence systems, negative valence systems, cognitive systems, systems for social process, arousal/modulatory systems, and sensorimotor systems, each divided into a number of constructs and subconstructs (e.g., negative valence systems includes constructs such as “acute threat,” “sustained threat,” and “loss”) that can be interrogated at different units of analysis, comprising: genes, molecules, cells, circuits, physiology, behavior, self-reports, and paradigms.

Within each unit, elements for potential investigation are identified; for example, “avoidance” as a unit of behavior relevant to the construct of acute threat. RDoC builds on previous, albeit more localized, efforts to identify transdiagnostic processes such as Harvey and colleagues’ ground-breaking 2004 book (Harvey, Watkins, Mansell, & Shafran, 2004) that identified a portfolio of transdiagnostic cognitive processes (e.g., selective attention, overgeneral memory, repetitive negative thinking) that manifest in both clinical (i.e., diagnosed) and nonclinical samples, and that had been implicated across four or more disorders (see Supplementary Table S2). This initiative formed the basis for a swathe of process-driven interventions either focused on individual processes that have a transdiagnostic reach, for example memory specificity training (Werner-Seidler et al., 2018), attentional bias modification (MacLeod & Clarke, 2015), interpretive bias modification for specific processes such as repetitive negative thinking (RNT; Hirsch et al., 2020), attentional control training for cognitive anxiety sensitivity treatment (Allan, Albanese, Judah, & Schmidt, 2020), or that encompass a broader set of processes within the context of established intervention paradigms, for example rumination focused CBT (RfCBT; Watkins, 2016) or augmented depression therapy (ADepT; Dunn et al., 2019), along with process-based CBT more broadly (Hayes & Hofmann, 2018).

The rationale for RDoC (and related efforts) is to provide a framework for research into the biopsychosocial substrates of mental ill health. This ambition is deeply laudable but it also raises an interesting and challenging question—how do we decide what constructs to include? RDoC identifies a number of inclusion criteria. Most central is the judgment that a construct is “relevant” to understanding some aspect of “psychopathology” (Brent et al., 2018; Morris & Cuthbert, 2012). But, of course, this judgment rests on elucidating responses to two issues at the heart of the discipline—how we define “relevance” and how we characterize psychopathology.

The characterization of psychopathology is admirably open-ended in the RDoC. They argue persuasively that the priority is to facilitate the evaluation of hypothesis-driven research questions that pertain to clinically significant symptoms or dysfunction. It is therefore as legitimate to look at symptom dimensions (for instance, at any level of a hierarchical framework such as HiTOP) as to consider clusters of classically defined *DSM* disorders (Cuthbert, 2015), or even presumably single disorders.

With respect to “relevance,” RDoC characterizes constructs as relevant to psychopathology as a function of “. . . *increasing deregulation in functionality that can be construed as falling at one extreme or the other of the normal distribution*” (Cuthbert, 2015; p. 95). This raises the question of what constitutes a dysfunctional (“deregulated in functionality”) process. It seems clear that, despite RDoC’s claims, falling at an extreme of the normal distribution is not always itself sufficient without some additional notion of impairment or maladaptiveness (Wakefield, 2014). For instance, extreme avoidance of threat is adaptive when in a highly threatening environment such as a combat zone but arguably becomes maladaptive in low-threat contexts (Berenbaum, 2013). Furthermore, even if one-tailed of the normal distribution does reflect dysfunctionality, that does not mean that both tails do; for instance, poor working memory may be dysfunctional, but at the other extreme it is unlikely to be a source of concern, however excellent it is (Cuthbert & Insel, 2013).

Finally, constructs do not even have to fall at extremes of the distribution under any circumstances to be relevant to psychopathology. The first two RDoC domains—negative and positive valence systems—combine identified processes (e.g., attention) that acting upon particular content (positive or negative information) to organize constructs where it is perhaps easier to see how extremities of the distribution of operation may be dysfunctional (although see the attentional bias example below). However, other RDoC domains—cognitive systems, social processes, and so forth—eschew content entirely. This means that processes within these domains could be operating entirely normatively *but on dysfunctional content* and it is the latter that confers the “relevance” to psychopathology. Indeed, this interaction between normative processes and difficult content is a cornerstone of most cognitive models of emotional disorder (Beck, Rush, Shaw, & Emery, 1979; Power & Dalgleish, 2015). For instance, having an entirely normative ability to understand one’s own agency (an RDoC element) while trapped in a coercive relationship where one is disempowered, reflects the operation of a normative process in a dysfunctional context. Here, arguably, the process-content interplay of self-perceived disempowerment is profoundly relevant to understanding the mechanisms underlying any psychopathological response.

Relatedly, different RDoC constructs, each operating within normative ranges, may interact with each other to generate disturbed or dysfunctional responses relevant to psychopathology; for instance, for some individuals (those with depression or vulnerable to manic episodes) rewarding experiences might be experienced as threatening, leading to avoidance behavior, without any abnormalities in the functioning of the positive or negative valence systems themselves. Finally, RDoC constructs operating within the normative range may nevertheless be relevant to understanding risk or vulnerability to mental health problems. For example, those processes may represent diatheses such that encountering a particular context will precipitate the onset of difficulties.

If we do set aside the criterion that the processing constructs themselves have to be operating “abnormally” (i.e., at extreme ends of the normal distribution), this means that we are left with no obvious way to construe a given construct as problematic or dysfunctional. Similarly, it becomes impossible to apply cut-offs to define, for example, mild, moderate, or severe levels of dysfunction. Indeed, once we relax the “abnormality criterion,” it becomes difficult to rule out almost any process as relevant to psychopathology due to the systemic nature of the majority of mental health problems where the multiplicity of mental operations are implicated. If this is the case, then initiatives such as RDoC will *de facto* eventually spawn frameworks for conceptualizing the different levels of processes involved in human mental life, rather than anything specific to psychopathology. This would arguably edge RDoC and like systems toward unfalsifiability in terms of their stated aims as it would be unclear what would constitute an irrelevant process.

Part of this problem lies with the boundary conditions around the concept of “relevance” itself. Across the developmental trajectory that RDoC promotes (Casey, Oliveri, & Insel, 2014), there will be countless factors and processes associated with either the outcome of mental health problems or the risk thereof. All of these constructs are therefore “relevant” in the broadest sense but without appropriate theories about the etiology of particular difficulties, it is hard to distinguish processes that are proximally causal from those that are either distally causal or merely associated with mental health problems. For example, an attentional bias toward threat (an RDoC subconstruct) seems clearly associated with many anxiety- and stressor-related conditions (Mogg, Mathews, & Eysenck, 1992), and has been shown (in experimental manipulations) to elevate feelings of anxiety (MacLeod, Rutherford, Campbell, Ebsworthy, & Holker, 2002). This has led to a plethora of ‘cognitive bias modification’ interventions to retrain the attentional system away from threat as a putative treatment for anxiety (MacLeod & Clarke, 2015). However, to date these approaches appear to have limited longer-term impact on anxiety symptoms (Cristea, Kok, & Cuijpers, 2015; Jones & Sharpe, 2017). Why might this be? Cognitive theories of anxiety disorders would argue that much of the attentional bias is driven by deep-rooted beliefs or models of the world that confer danger on the stimuli that are attended to (Bar-Haim et al., 2007; Wells & Matthews, 1994) and so the deep cause of the anxiety problems is the possession of such beliefs. Consequently, retraining attention away from threatening stimuli will only have short-lasting effects on anxiety as relatively quickly the underlying belief representations will simply recalibrate the attentional system to be alert for the specified dangers once more. An analogy would be the common experience of preferentially noticing other cars on the road that are the same as, say, one’s own red Jeep. You could train your attention system away from red Jeeps and toward blue Land Rovers, but as long as you continue to drive about town in your Jeep it is most likely that you’ll soon be noticing other Jeeps again. However, if you traded in the Jeep and acquired a new blue Land Rover, then the attentional bias for Jeeps would eventually fade away. It is the ownership of the Jeep that is central here and the attentional bias for Jeeps simply flows from that.

Aspiring to a deeper understanding of causality when considering the role of transdiagnostic biopsychosocial processes raises another question. How do we distinguish causal processes that are relevant to developing clinical interventions from those that are perhaps less helpful? Wakefield (2014) uses the analogy of a plane crash. Both a failed engine, for example, and gravity are causally involved in the crash, but efforts to understand what went wrong and how to use those insights to prevent future crashes are better focused on the engine’s vulnerabilities rather than gravity. To develop interventions, therefore, we need to focus on causal processes that are tractable and malleable through intervening, as opposed to those processes that are relatively immutable. Harvey, Murray, Chandler, and Soehner (2011) make a related point distinguishing between processes that are “descriptively transdiagnostic” (i.e., present in a range of diagnoses), from those that are “mechanistically transdiagnostic” (i.e., reflecting a causal, functional mechanism).

Finally, other thorny issues pertaining to process causality that present a challenge for how we map processes onto mental ill health presentations concern how to make sense of multifinality—whereby the same causal pathway appears to lead to a range of different mental health difficulties—and equifinality whereby multiple causal pathways appear to give rise to the same outcome (Cicchetti & Rogosch, 1996).

As with the pioneering approaches to transdiagnostic nosology, RDoC seeks to maintain a theoretical neutrality. This reticence of RDoC to align itself with any given theoretical paradigm is understandable, and echoes the philosophy of the post-*DSM–III* approach in the *DSM*, but it is questionable whether any such endeavor that aspires to address questions of causality and mechanism with a view to capitalizing on those insights to develop novel process-based interventions can really succeed without clear theoretical principles that provide a basis for sorting the mechanistic wheat from the chaff, even if those theories will inevitably require refinement over time (Mansell, 2019).

## Transdiagnostic Clinical Approaches

Transdiagnostic clinical interventions that “. . . *apply the same underlying treatment principles across mental disorders, without tailoring the protocol to specific diagnoses*” (McEvoy, Nathan, & Norton, 2009, p. 21), have evolved over the last 20 years in parallel with, but mostly independently from, the developments in transdiagnostic nosology and process-based science outlined above. The post-*DSM–III* impetus has come from two sobering aspects of clinical reality. First, the aforementioned rampant comorbidity and pervasive heterogeneity of clinical presentations, which ensures that tailored single diagnosis-led protocols often struggle to address the range of problems with which clients present. Second, the real need to do more to improve the effectiveness of our psychological (and pharmacological) interventions, where even our best available diagnosis-led treatments only achieve clinical recovery for 40–70% of patients, depending on their primary diagnosis, with people suffering complex comorbid conditions faring significantly worse (Moses & Barlow, 2006). The primary rationale for transdiagnostic clinical interventions therefore is that they should better address the heterogeneity and comorbidity that is the modal presentation in real-world services, thus delivering improved clinical effectiveness (Ellard, Fairholme, Boisseau, Farchione, & Barlow, 2010).

Transdiagnostic psychological interventions fall into two broad categories (Meidlinger & Hope, 2017; Sauer-Zavala et al., 2017). Universal interventions such as the *Unified Protocol for Transdiagnostic Treatment of Emotional Disorders* (Barlow et al., 2010) promote a one-size-fits-all approach where all clients receive the same set of therapeutic elements that have been carefully selected to have the broadest applicability across diagnoses. The fact that practitioners can adopt such universal approaches without modification for a wide range of mental health conditions has a number of practical advantages. Clinical application is easier as there is no bespoke selection of intervention elements. Clinical training is also facilitated thus minimizing barriers to dissemination, compared with standard training models that involve learning multiple treatment protocols for different disorders (Steele et al., 2018). However, a drawback of universal approaches is that their very universality precludes tailored selection of treatment elements to the particular presentations of individual clients.

In contrast, modular approaches, such *The Modular Approach to Therapy for Anxiety, Depression, Trauma, or Conduct Problems* (MATCH-ADTC) for children (Chorpita, Daleiden, & Weisz, 2005b) and the *Shaping Healthy Minds* protocol targeting the same range of problems in adults (Black et al., 2018), comprise sets of evidence-based self-contained functional units (therapy modules) that can operate independently and be delivered flexibly, so that module selection and order are tailored to the needs of each client. For example, Evans et al. (2020) present the potential application of the MATCH-ADTC protocol for children with severe irritability. Modular interventions are more challenging to deliver than universal protocols, requiring algorithms to match module delivery to the separate requirements of each client, but this in principle comes with a better goodness-of-fit between the therapy and the individual clinical presentation (see Supplementary Table S3 for an outline of the intervention components in selected universal and modular transdiagnostic approaches).

Transdiagnostic interventions also vary in their breadth of application (Craske, 2012). Some focus on a relatively narrow range of clinical presentations such as Fairburn’s transdiagnostic approach to eating disorders (Fairburn, Cooper, & Shafran, 2003), whereas other approaches have a much broader purview, for example Transdiagnostic Group CBT for anxiety (Norton, Hayes, & Hope, 2004). There is an obvious trade-off, with greater breadth meaning that the intervention is perhaps less tailored for the specifics of any individual constellation of symptoms or disorder.

Numerous systematic reviews and meta-analyses (Andersen, Toner, Bland, & McMillan, 2016; Newby, McKinnon, Kuyken, Gilbody, & Dalgleish, 2015; Newby, Twomey, Yuan Li, & Andrews, 2016; Pearl & Norton, 2017; Păsărelu et al., 2017; Reinholt & Krogh, 2014) broadly support the equivalence or superiority of transdiagnostic psychological treatments over comparison or control interventions (comprising either a diagnosis-specific intervention control, treatment-as-usual, or a waitlist control; summarized in Supplementary Table S4). As with all interventions, the nature of the comparison condition, and of the trial design more generally, are critical to evaluating efficacy and effectiveness. For example, one would not necessarily expect transdiagnostic interventions to be more efficacious than single-diagnosis-led protocols for what one might characterize as the primary clinical (diagnostic) problem that the client presents with. Going forward, it therefore seems essential that the field develops consensus criteria for what would constitute compelling evidence that a transdiagnostic intervention is successful.

### What Would Constitute Success for a Transdiagnostic Intervention?

Ever since Saul Rosenzweig pronounced Lewis Carroll’s Dodo bird verdict—“*Everybody has won and all must have prizes*”—on the field of psychotherapy, it has generally been accepted that a significant proportion of the variance in psychotherapy effectiveness can be attributed to “common factors” (Cuijpers, Reijnders, & Huibers, 2019; Norcross & Wampold, 2011), such as patient expectations, structured regular sessions and the client–therapist relationship, that are shared by all psychotherapies (Chambless, 2002; Luborsky, Singer, & Luborsky, 1975). In addition to common factors, there are shared principles across interventions within each psychological therapy model—what we shall call “common processes.” For example, CBT includes common processes that are shared by most, if not all, diagnosis-led CBT protocols (Hayes & Hofmann, 2018; see Supplementary Table S2). The combined contribution of these common factors and common processes to therapeutic outcomes has clear implications for understanding and developing any new interventions including transdiagnostic protocols, where arguably such components deliver much of the clinical impact. Additional efficacy is then delivered by specific therapy ingredients tailored to particular clinical presentations (e.g., depression, panic)—what we shall call for the present purposes “diagnosis-specific processes.” Transdiagnostic protocols are predicated on the assumption that they contain such diagnosis-specific therapy ingredients for *multiple* disorders, whereas single diagnosis-led interventions heavily focus on diagnosis-specific processes only for the primary disorder that they target.

**Figure 2 fig2:**
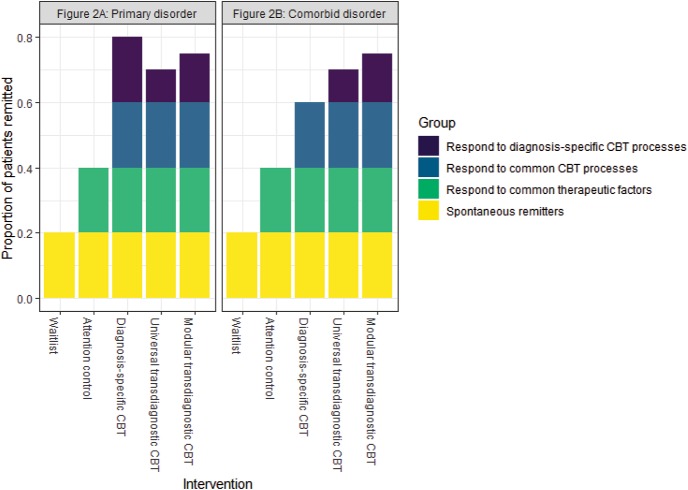
Predicted treatment responses (proportions of patients remitted) to diagnosis-specific and transdiagnostic interventions, and relevant control conditions. Treatment nonresponders are not included in the figure. CBT = cognitive-behavior therapy.

In Figure 2, inspired by DeRubeis, Gelfand, German, Fournier, and Forand (2014), to understand the impact of these different processes on clinical outcomes, we consider the putative contributions to therapeutic efficacy (using CBT as an exemplar) of common factors, common processes, and diagnosis-specific processes, for hypothetical patients presenting with comorbid clinical problems. In other words, where there is an identifiable primary problem or disorder (e.g., depression) that would inform choice of a diagnosis-led treatment, alongside comorbid difficulties (e.g., panic, social anxiety) that arguably also require intervention.[Fig-anchor fig2]

Figure 2 therefore compares a hypothetical diagnosis-specific CBT intervention targeted at a “primary diagnosis”^3^ against, both universal and modular hypothetical transdiagnostic CBT interventions, which both target primary and comorbid diagnoses equally. We compare all three of these active CBT interventions against both a placebo- or attention-control condition (i.e., something that includes common factors but not common processes or diagnosis-specific processes), and a wait-list control that lacks common factors, and any active processes.

For all of these five interventions and comparison conditions, there will be a proportion of patients who do not respond, showing no remediation of symptoms, either because their problems are intractable or because they are unable to engage with the interventions for practical or psychological reasons.^4^ These nonresponders aside, Figures 2A and 2B illustrate the predicted proportion of therapeutic response, for the remaining patients, offered by the different hypothetical intervention protocols for the primary diagnosis and comorbid disorder(s), respectively.

The figure illustrates a number of important points. First, for all conditions there will be a proportion of patients who spontaneously remit irrespective of the help they receive. Second, both diagnosis-specific and transdiagnostic CBT interventions should outperform wait-list and attention-control comparison conditions for both primary and comorbid diagnoses due to the common CBT processes that the three active interventions share and which the control conditions lack—a conclusion broadly supported by the literature (e.g., Newby et al., 2015; Supplementary Table S4). As a consequence, the lion’s share of the variance in treatment response would be predicted to be comparable across these three active interventions and is accounted for by the combination of therapeutic nonresponse, spontaneous clinical remission, or the impact of shared common factors and common processes. Finally, as a result, the potential differences between the three active interventions are predicted to not only be small but also likely to differ as a function of whether we are considering the primary disorder or the comorbid difficulties with diagnosis-specific interventions likely to be more efficacious for the former, and transdiagnostic approaches more efficacious for the latter.

Of course, the figure is hypothetical but if the broad principles are true, this has a number of key implications for how we evaluate transdiagnostic clinical interventions:

1 The critical comparison for evaluating emergent transdiagnostic interventions should be against current gold-standard diagnosis-led approaches (Sauer-Zavala et al., 2017; Watkins, 2018). This has rarely been the case, to date (Supplementary Table S4). However, when considering this critical comparison, it is likely that for identified primary disorders, transdiagnostic approaches will actually be *less efficacious* than established diagnosis-specific treatments. Furthermore, for any comorbid disorders, the shared presence of both common factors and common processes across both diagnosis-specific and transdiagnostic interventions means that, although one would expect an efficacy advantage for transdiagnostic interventions due to their inclusion of a broader portfolio of treatment elements targeting diagnosis-specific processes, the effect size of such an advantage is likely to be small (Craske, 2012).
2 This has significant implications for clinical trial methodology within the transdiagnostic domain. First, evaluated clinical outcomes in trials will need to encompass not only the primary disorder but also any comorbid difficulties, if the benefits of transdiagnostic approaches are to be revealed. Second, novel trial designs will be required; for instance, hybrid designs comprising a noninferiority component for comparing the transdiagnostic intervention relative to the diagnosis-led treatment for a primary disorder, alongside an additional superiority design component (favoring the transdiagnostic intervention) for the comorbid problems. Finally, even for this superiority component, hypothesized effect sizes are likely to be small and consequently requisite sample sizes will need to be large to provide adequate statistical power.
3 The likely small differences in efficacy between transdiagnostic approaches and established diagnosis-led interventions highlight the need for any proper evaluation of the utility of the transdiagnostic approach to extend beyond simple efficacy (Meidlinger & Hope, 2017; Watkins, 2018). As already noted, transdiagnostic interventions in principle confer significant advantages in terms of training and dissemination^5^ and thus implementation within health care systems. This means that, in the broader scheme, it is likely more cost-effective to disseminate and implement a smaller set of transdiagnostic approaches than a much larger set of diagnosis-specific interventions. The challenge for clinical trialists is to capture these benefits in their evaluations.

### Linking Transdiagnostic Interventions to Transdiagnostic Processes

Even though many transdiagnostic interventions have an identified suite of therapeutic processes at their core—whether they be common factors, common processes, or diagnosis-specific processes for multiple conditions—empirically demonstrating that these processes genuinely represent the mechanisms of therapeutic action has proved a challenge, as it has for the field more generally (Cuijpers et al., 2019). One way forward is for embedded process-outcome studies that are theoretically informed and suitably statistically powered to become mandated for all large-scale clinical trials. Such studies should eschew simple correlational models, include a broad array of potential mediators, pay close attention to both dose-response relationships and temporal associations between mediating processes and outcomes, and be combined with adjunctive clinical science involving the experimental manipulation of potential mediators (Lorenzo-Luaces & DeRubeis, 2018).

Care also needs to be taken that putative mediating processes are properly activated or engaged within the assessment protocols as opposed to being assessed “cold.” For example, if a treatment reduces symptoms of emotional distress, such a cold posttreatment assessment might show that dysfunctional thinking patterns typically associated with that distress are similarly mitigated. However, an experimental mood induction designed to activate patterns of “hot” cognition may reveal that such thinking styles are simply latent and easily reactivated following a downturn in mood (Kuyken et al., 2010; Teasdale, 1988).

We also need to move beyond the examination of between-participants nomothetic processes (e.g., differences in the therapeutic alliance) to include consideration of key idiographic processes that dynamically shift across time within individuals (e.g., how the alliance changes across the course of therapy). Modeling individual processes across time, as well as general and shared processes across individuals, can help better understand dynamic patterns in context to identify maintenance mechanisms and treatment targets (see Woods et al., 2020).

Perhaps a more fundamental process-related concern is the translational chasm between the process-relevant discovery science outlined above and intervention development. Almost all current evidence-based psychological interventions, with the possible exception of behavior therapy, have their roots in the clinic rather than the science laboratory. This is as true of transdiagnostic approaches where, to date, the vast majority of clinical advances have emerged through the distillation of existing evidence-based treatment elements (Chorpita, Daleiden, & Weisz, 2005a) from established diagnosis-led protocols—predominantly CBT. Very few, if any, transdiagnostic clinical applications have either emerged out of, or benefited from, advances in fundamental underlying transdiagnostic biopsychosocial process research, such as RDoC. Bridging this translational gap is a major challenge.

## Five Challenges for Transdiagnostic Science

Having sought to summarize and evaluated the current state of transdiagnostic science, we now distil five challenges that require our collective attention if the field is to progress.

1 Development of relevant theory: Much of the transdiagnostic field rests on atheoretical foundations, especially work on core processes and nosology. As we have argued, although the rationale for this is understandable, it is not clear how the translational potential of this work into novel clinical approaches can be fully harvested without appropriate theoretical frameworks to guide it. A key challenge therefore is to develop metatheoretical models that transcend the established diagnosis-specific frameworks that currently drive clinical translation (e.g., Mansell, 2019; Power & Dalgleish, 2015).
2 A focus on mental content: The vast majority of clinical discourse between practitioners and patients concerns the *contents* of thoughts, beliefs, and assumptions about the world—negative thoughts, ruminative narratives, dysfunctional core beliefs, toxic mental images, and so on. Engaging with the nature of such mental content is consequently a cornerstone of major therapeutic models within the field, such as CBT. However, much of the work on fundamental transdiagnostic processes (e.g., within RDoC) is agnostic to the content of the material being processed (with important exceptions e.g., RDoC’s “negative valence systems”). It seems deeply unlikely that a comprehensive understanding of how dysfunctional processes impinge on mental health will be achieved until the critical interactions between mental processes and mental content are appropriately understood and elucidated.
3 Setting diagnoses aside: We have drawn a distinction between “soft” and “hard” transdiagnostic approaches, with the former retaining a diagnostic framework yet seeking commonalities across diagnoses, and the latter more radically seeking to replace diagnoses with alternative formulations. We submit that a genuine alternative to the established diagnostic nosologies will only emerge once harder transdiagnostic paradigms are properly embraced.^6^
4 Developing fit-for-purpose research methodology: The current prototypical superiority clinical trial design that focuses on a single primary outcome is arguably unsuitable for the proper evaluation of transdiagnostic interventions. Large-scale hybrid designs (incorporating noninferiority components), or other novel approaches (Blackwell, Woud, Margraf, & Schönbrodt, 2019), with multiple coprimary outcomes, and appropriate assessment of cost-effectiveness, are indicated. Such trials should also include mandated embedded process-outcome evaluations to address questions of what works for whom, what are the mechanisms of clinical change, and how intraindividual (idiographic) patterns of relationships between symptoms and processes change across time.
5 Prioritizing joined-up thinking: The diagnostic paradigm is long-lived and has an iron grip on current research and clinical practice in mental health. The majority of mental health care systems across the world have diagnoses at the heart of assessment, clinical practice, and health care structure. In terms of clinical research, localized research efforts are less likely to prise diagnostic fingers free than larger-scale consortia such as RDoC and HiToP, representing the collaborative efforts of many international scientists from multiple disciplines. Joining and supporting these consortia, while of course seeking to engage with any scientific and philosophical differences one might have with them from within, is therefore to be highly encouraged. Implementing transdiagnostic approaches into clinical practice will prove more of a challenge and rests crucially on the development of robust transdiagnostic assessment instruments and interventions that can supplant their diagnostic counterparts. The clinical jury remains out.

## Preview of Articles in This Special Section

We have gathered a collection of original articles instantiating the key themes reviewed above. They provide an exciting snapshot of the plethora of research focusing on transdiagnostic approaches to common mental health problems.

In the domain of nosology, Schweizer et al. (2020) have applied a bifactor approach to internalizing and externalizing dimensions of cognitive and general psychopathology risk for early adolescents, younger youth, and older youth.

In the process domain, Allan et al. (2020) provide an excellent example of integrating process measures into clinical trials. They describe a multimethod investigation on how individual differences in a key process variable (attentional control) modulate clinical response in the context of a brief transdiagnostic treatment for anxiety and depression. Hirsch et al. (2020) describe an example of clinical translation from basic science on negative interpretation bias. They found that augmenting interpretation training for repetitive negative thinking with positive outcome generation and imagery facilitated more positive interpretations, reduced negative intrusions after training, and reduced trait rumination. Woods et al. (2020) modeled individual processes as well as general and shared processes across individuals with borderline personality disorder, finding a significant degree of heterogeneity in interpersonal and affective domains.

In the clinical domain, Evans, Weisz, Carvalho, Garibaldi, Bearman, Chorpita, and The Research Network for Youth Mental Health (2020) report on the evaluation of a modular, transdiagnostic, cognitive–behavioral intervention compared with standard manualized treatments and usual care for treating youth with severe irritability across multiple outcomes, informants, and measurement schedules. Finally, Käll et al. (2020) describe the use of a distillation and matching approach to identifying standardized evidence-supported interventions to reduce loneliness, by coding for the presence of cognitive–behavioral practice elements and maintaining mechanisms.

## Supplementary Material

10.1037/ccp0000482.supp

## Footnotes

1 For the *DSM–5* there were originally much more radical plans to incorporate dimensionality, especially within the personality disorders, but these did not come to fruition (Kraemer, 2007).

2 These concerns about heterogeneity within diagnoses (see above) and incomplete capture of the prototypical symptom presentations of different putative disorders obviously pull in different directions.

3 We have used the terminology of primary and comorbid diagnoses or disorders for ease of illustration but in principle the same points can be made for symptom clusters or dimensions.

4 It is to be hoped that it is possible that precision medicine initiatives (e.g., Cohen & DeRubeis, 2018) will match individuals to interventions better suited to their specific difficulties to reduce the proportion of non-responders, although the clinical benefits of this approach are yet-to-be realized.

5 It is important to note that this is not the same as saying that training and implementation are straightforward in the transdiagnostic domain. We know that training and dissemination for even more straightforward healthcare approaches to mental health difficulties can prove very challenging (Proctor et al., 2009). Rather we are saying that it should be relatively better than the training and dissemination challenge posed when there are a multitude of similar and overlapping approaches to implement.

6 It may be that the case for a “hard” transdiagnostic line is more compelling for common mental health problems (National Collaborating Centre for Mental Health, 2011)—currently categorized as mood, anxiety, and stressor-related disorders—than for other constellations of difficulties.

## References

[c1] *Allan N. P., Albanese B. J., Judah M. R., & Schmidt N. B. (2020). A multi-method investigation of the impact of attentional control on a brief intervention for anxiety and depression. Journal of Consulting and Clinical Psychology, 88, 212–225. 10.1037/ccp0000484 32068423

[c2] American Psychiatric Association (1952). Diagnostic and statistical manual of mental disorders (1st ed.). Washington, DC: Author.

[c3] American Psychiatric Association (2013). Diagnostic and statistical manual of mental disorders, 5th edition (DSM–5). Washington, DC: Author.

[c4] Andersen P., Toner P., Bland M., & McMillan D. (2016). Effectiveness of transdiagnostic cognitive behaviour therapy for anxiety and depression in adults: A systematic review and meta-analysis. Behavioural and Cognitive Psychotherapy, 44, 673–690. 10.1017/S1352465816000229 27301967

[c146] Baker J. T., Dillon D. G., Patrick L. M., Roffman J. L., Brady R. O., Pizzagalli D. A., . . . Holmes A. J. (2019). Functional connectomics of affective and psychotic pathology. Proceedings of the National Academy of Sciences, 116, 9050–9059. 10.1073/pnas.1820780116 PMC6500110 30988201

[c5] Bar-Haim Y., Lamy D., Pergamin L., Bakermans-Kranenburg M. J., & van IJzendoorn M. H. (2007). Threat-related attentional bias in anxious and nonanxious individuals: A meta-analytic study. Psychological Bulletin, 133, 1–24. 10.1037/0033-2909.133.1.1 17201568

[c6] Barlow D. H., Ellard K. K., Fairholme C. P., Farchione T. J., Boisseau C. L., Allan L. B., & Ehrenreich-May J. T. (2010). Unified protocol for transdiagnostic treatment of emotional disorders: Workbook (1st ed.). Oxford, UK: Oxford University Press 10.1093/med:psych/9780199772667.001.0001

[c7] Beck A. T., Rush A. J., Shaw B. F., & Emery G. (1979). Cognitive therapy of depression. New York, NY: Guilford Press.

[c8] Berenbaum H. (2013). Classification and psychopathology research. Journal of Abnormal Psychology, 122, 894–901. 10.1037/a0033096 24016025

[c9] Black M., Hitchcock C., Bevan A. O., Leary C., Clarke J., Elliott R., . . . Dalgleish T. (2018). The HARMONIC trial: Study protocol for a randomised controlled feasibility trial of Shaping Healthy Minds-a modular transdiagnostic intervention for mood, stressor-related and anxiety disorders in adults. British Medical Journal Open, 8, e024546 10.1136/bmjopen-2018-024546 PMC6078277 30082367

[c10] Blackwell S. E., Woud M. L., Margraf J., & Schönbrodt F. D. (2019). Introducing the leapfrog design: A simple Bayesian adaptive rolling trial design for accelerated treatment development and optimization. Clinical Psychological Science, 7, 1–22. 10.1177/2167702619858071

[c11] Blashfield R. (1984). The classification of psychopathology: Neo-kraepelinian and quantitative approaches. Berlin, Germany: Springer 10.1007/978-1-4613-2665-6

[c12] Blashfield R. K., Keeley J. W., Flanagan E. H., & Miles S. R. (2014). The cycle of classification: *DSM-I* through *DSM–5*. Annual Review of Clinical Psychology, 10, 25–51. 10.1146/annurev-clinpsy-032813-153639 24679178

[c13] Borsboom D. (2017). A network theory of mental disorders. World Psychiatry, 16, 5–13. 10.1002/wps.20375 28127906 PMC5269502

[c14] Borsboom D., & Cramer A. O. (2013). Network analysis: An integrative approach to the structure of psychopathology. Annual Review of Clinical Psychology, 9, 91–121. 10.1146/annurev-clinpsy-050212-185608 23537483

[c15] Brent D., Miller G. A., Noronha J., Arean P., Barch D. A., Heimer H., . . . Vaidyanathan U. (2018). RDoC changes to the matrix CMAT workgroup update: Proposed positive valence domain revisions. Bethesda, MD: National Institute of Mental Health Retrieved from https://www.nimh.nih.gov/about/advisory-boards-and-groups/namhc/reports/rdoc-changes-to-the-matrix-cmat-workgroup-update-proposed-positive-valence-domain-revisions.shtml#Introduction

[c16] Brown T. A., Campbell L. A., Lehman C. L., Grisham J. R., & Mancill R. B. (2001). Current and lifetime comorbidity of the *DSM–IV* anxiety and mood disorders in a large clinical sample. Journal of Abnormal Psychology, 110, 585–599. 10.1037/0021-843X.110.4.585 11727948

[c17] Buckholtz J. W., & Meyer-Lindenberg A. (2012). Psychopathology and the human connectome: Toward a transdiagnostic model of risk for mental illness. Neuron, 74, 990–1004. 10.1016/j.neuron.2012.06.002 22726830

[c18] Bystritsky A., Nierenberg A. A., Feusner J. D., & Rabinovich M. (2012). Computational non-linear dynamical psychiatry: A new methodological paradigm for diagnosis and course of illness. Journal of Psychiatric Research, 46, 428–435. 10.1016/j.jpsychires.2011.10.013 22261550

[c19] Casey B. J., Oliveri M. E., & Insel T. (2014). A neurodevelopmental perspective on the research domain criteria (RDoC) framework. Biological Psychiatry, 76, 350–353. 10.1016/j.biopsych.2014.01.006 25103538

[c20] Caspi A., Houts R. M., Belsky D. W., Goldman-Mellor S. J., Harrington H., Israel S., . . . Moffitt T. E. (2014). The p Factor: One general psychopathology factor in the structure of psychiatric disorders? Clinical Psychological Science, 2, 119–137. 10.1177/2167702613497473 25360393 PMC4209412

[c21] Chambless D. (2002). Beware the Dodo bird: The dangers of overgeneralization. Clinical Psychology: Science and Practice, 9, 13–16. 10.1093/clipsy.9.1.13

[c23] Chorpita B. F., Daleiden E. L., & Weisz J. R. (2005a). Identifying and selecting the common elements of evidence based interventions: A distillation and matching model. Mental Health Services Research, 7, 5–20. 10.1007/s11020-005-1962-6 15832690

[c22] Chorpita B. F., Daleiden E. L., & Weisz J. R. (2005b). Modularity in the design and application of therapeutic interventions. Applied & Preventive Psychology, 11, 141–156. 10.1016/j.appsy.2005.05.002

[c147] Cicchetti D., & Rogosch F. A. (1996). Equifinality and multifinality in developmental psychopathology. Development and Psychopathology, 8, 597–600. 10.1017/S0954579400007318

[c24] Clark L. A., Cuthbert B., Lewis-Fernández R., Narrow W. E., & Reed G. M. (2017). Three approaches to understanding and classifying mental disorder: ICD-11, *DSM–5*, and the National Institute of Mental Health’s Research Domain Criteria (RDoC). Psychological Science in the Public Interest, 18, 72–145. 10.1177/1529100617727266 29211974

[c25] Cohen Z. D., & DeRubeis R. J. (2018). Treatment selection in depression. Annual Review of Clinical Psychology, 14, 209–236. 10.1146/annurev-clinpsy-050817-084746 29494258

[c26] Compton W. M., & Guze S. B. (1995). The neo-Kraepelinian revolution in psychiatric diagnosis. European Archives of Psychiatry and Clinical Neuroscience, 245, 196–201. 10.1007/BF02191797 7578281

[c27] Cosgrove L., Krimsky S., Vijayaraghavan M., & Schneider L. (2006). Financial ties between *DSM–IV* panel members and the pharmaceutical industry. Psychotherapy and Psychosomatics, 75, 154–160. 10.1159/000091772 16636630

[c28] Craddock N., & Owen M. J. (2010). The Kraepelinian dichotomy—Going, going . . . but still not gone. The British Journal of Psychiatry, 196, 92–95. 10.1192/bjp.bp.109.073429 20118450 PMC2815936

[c29] Craig T. K. J., & Boardman A. P. (1997). ABC of mental health. Common mental health problems in primary care. British Medical Journal, 314, 1609–1612. 10.1136/bmj.314.7094.1609 9186176 PMC2126815

[c30] Cramer A. O., Waldorp L. J., van der Maas H. L., & Borsboom D. (2010). Complex realities require complex theories: Refining and extending the network approach to mental disorders. Behavioral and Brain Sciences, 33, 178–193. 10.1017/S0140525X10000920

[c31] Craske M. G. (2012). Transdiagnostic treatment for anxiety and depression. Depression and Anxiety, 29, 749–753. 10.1002/da.21992 22949272

[c32] Cristea I. A., Kok R. N., & Cuijpers P. (2015). Efficacy of cognitive bias modification interventions in anxiety and depression: Meta-analysis. The British Journal of Psychiatry, 206, 7–16. 10.1192/bjp.bp.114.146761 25561486

[c33] Cuijpers P., Reijnders M., & Huibers M. J. H. (2019). The role of common factors in psychotherapy outcomes. Annual Review of Clinical Psychology, 15, 207–231. 10.1146/annurev-clinpsy-050718-095424 30550721

[c34] Cuthbert B. N. (2015). Research domain criteria: Toward future psychiatric nosologies. Dialogues in Clinical Neuroscience, 17, 89–97. 25987867 10.31887/DCNS.2015.17.1/bcuthbert PMC4421905

[c35] Cuthbert B. N., & Insel T. R. (2013). Toward the future of psychiatric diagnosis: The seven pillars of RDoC. BMC Medicine, 11, 126 10.1186/1741-7015-11-126 23672542 PMC3653747

[c36] Derubeis R. J., Gelfand L. A., German R. E., Fournier J. C., & Forand N. R. (2014). Understanding processes of change: How some patients reveal more than others-and some groups of therapists less-about what matters in psychotherapy. Psychotherapy Research, 24, 419–428. 10.1080/10503307.2013.838654 24219275 PMC4210369

[c37] Dunn B. D., Widnall E., Reed N., Owens C., Campbell J., & Kuyken W. (2019). Bringing light into darkness: A multiple baseline mixed methods case series evaluation of Augmented Depression Therapy (ADepT). Behaviour Research and Therapy, 120, 103418 10.1016/j.brat.2019.103418 31310929

[c39] Eaton N. R., Krueger R. F., Markon K. E., Keyes K. M., Skodol A. E., Wall M., . . . Grant B. F. (2013). The structure and predictive validity of the internalizing disorders. Journal of Abnormal Psychology, 122, 86–92. 10.1037/a0029598 22905862 PMC3755742

[c40] Ellard K. K., Fairholme C. P., Boisseau C. L., Farchione T., & Barlow D. H. (2010). Unified protocol for the transdiagnostic treatment of emotional disorders: Protocol development and initial outcome data. Cognitive and Behavioral Practice, 17, 88–101. 10.1016/j.cbpra.2009.06.002 PMC7986982 33762811

[c41] *Evans S., Weisz J. R., Carvalho A. C., Garibaldi P. M., Bearman S. K., & Chorpita B. F., & The Research Network for Youth Mental Health (2020). Effects of standard and modular psychotherapies in the treatment of youth with severe irritability. Journal of Consulting and Clinical Psychology, 88, 255–268. 10.1037/ccp0000456 32068426

[c42] Fairburn C. G., Cooper Z., & Shafran R. (2003). Cognitive behaviour therapy for eating disorders: A “transdiagnostic” theory and treatment. Behaviour Research and Therapy, 41, 509–528. 10.1016/S0005-7967(02)00088-8 12711261

[c43] Fichter M. M., Quadflieg N., Fischer U. C., & Kohlboeck G. (2010). Twenty-five-year course and outcome in anxiety and depression in the Upper Bavarian Longitudinal Community Study. Acta Psychiatrica Scandinavica, 122, 75–85. 10.1111/j.1600-0447.2009.01512.x 19922523

[c44] Frances A. (2009). A warning sign on the road to *DSM–5*: Beware of Its unintended consequences. The Psychiatric Times, 26, 8.

[c45] Fried E. I. (2017). The 52 symptoms of major depression: Lack of content overlap among seven common depression scales. Journal of Affective Disorders, 208, 191–197. 10.1016/j.jad.2016.10.019 27792962

[c46] Fried E. I., & Nesse R. M. (2015). Depression is not a consistent syndrome: An investigation of unique symptom patterns in the STAR*D study. Journal of Affective Disorders, 172, 96–102. 10.1016/j.jad.2014.10.010 25451401 PMC4397113

[c47] Funkhouser C. J., Correa K. A., Gorka S. M., Nelson B. D., Phan K. L., & Shankman S. A. (in press). The replicability and generalizability of internalizing symptom networks across five samples. Journal of Abnormal Psychology. 10.1037/abn0000496 PMC6980885 31829638

[c48] Galatzer-Levy I. R., & Bryant R. A. (2013). 636,120 ways to have posttraumatic stress disorder. Perspectives on Psychological Science, 8, 651–662. 10.1177/1745691613504115 26173229

[c49] Gellatly R., & Beck A. T. (2016). Catastrophic thinking: A transdiagnostic process across psychiatric disorders. Cognitive Therapy and Research, 40, 441–452. 10.1007/s10608-016-9763-3

[c50] Goekoop R., & Goekoop J. G. (2014). A network view on psychiatric disorders: Network clusters of symptoms as elementary syndromes of psychopathology. PLoS ONE, 9, e112734 10.1371/journal.pone.0112734 25427156 PMC4245101

[c51] Goodkind M., Eickhoff S. B., Oathes D. J., Jiang Y., Chang A., Jones-Hagata L. B., . . . Etkin A. (2015). Identification of a common neurobiological substrate for mental illness. Journal of the American Medical Association Psychiatry, 72, 305–315. 10.1001/jamapsychiatry.2014.2206 25651064 PMC4791058

[c52] Green J. G., McLaughlin K. A., Berglund P. A., Gruber M. J., Sampson N. A., Zaslavsky A. M., & Kessler R. C. (2010). Childhood adversities and adult psychiatric disorders in the national comorbidity survey replication I: Associations with first onset of *DSM–IV* disorders. Archives of General Psychiatry, 67, 113–123. 10.1001/archgenpsychiatry.2009.186 20124111 PMC2822662

[c53] Harvey A. G., Murray G., Chandler R. A., & Soehner A. (2011). Sleep disturbance as transdiagnostic: Consideration of neurobiological mechanisms. Clinical Psychology Review, 31, 225–235. 10.1016/j.cpr.2010.04.003 20471738 PMC2954256

[c54] Harvey A. G., Watkins E., Mansell W., & Shafran R. (Eds.). (2004). Cognitive behavioural processes across psychological disorders: A transdiagnostic approach to research and treatment. Oxford, UK: Oxford University Press 10.1093/med:psych/9780198528883.001.0001

[c55] Haslam N., Holland E., & Kuppens P. (2012). Categories versus dimensions in personality and psychopathology: A quantitative review of taxometric research. Psychological Medicine, 42, 903–920. 10.1017/S0033291711001966 21939592

[c56] Hayes S. C., & Hofmann S. G. (Eds.). (2018). Process-based CBT: The science and core clinical competencies of cognitive behavioral therapy. Oakland, CA: New Harbinger Publications.

[c57] Helzer J. E., Kraemer H. C., & Krueger R. F. (2006). The feasibility and need for dimensional psychiatric diagnoses. Psychological Medicine, 36, 1671–1680. 10.1017/S003329170600821X 16907995

[c58] Hicks B. M., Foster K. T., Iacono W. G., & McGue M. (2013). Genetic and environmental influences on the familial transmission of externalizing disorders in adoptive and twin offspring. Journal of the American Medical Association Psychiatry, 70, 1076–1083. 10.1001/jamapsychiatry.2013.258 23965950 PMC3790867

[c59] *Hirsch C. R., Krahé C., Whyte J., Bridge L., Loizou S., Norton S., & Mathews A. (2020). Effects of modifying interpretation bias on transdiagnostic repetitive negative thinking. Journal of Consulting and Clinical Psychology, 88, 226–239. 10.1037/ccp0000455 32068424

[c60] Hofmeijer-Sevink M. K., Batelaan N. M., van Megen H. J., Penninx B. W., Cath D. C., van den Hout M. A., & van Balkom A. J. (2012). Clinical relevance of comorbidity in anxiety disorders: A report from the Netherlands Study of Depression and Anxiety (NESDA). Journal of Affective Disorders, 137, 106–112. 10.1016/j.jad.2011.12.008 22240085

[c61] Hyman S. E. (2010). The diagnosis of mental disorders: The problem of reification. Annual Review of Clinical Psychology, 6, 155–179. 10.1146/annurev.clinpsy.3.022806.091532 17716032

[c62] Insel T. R. (2014). The NIMH Research Domain Criteria (RDoC) Project: Precision medicine for psychiatry. The American Journal of Psychiatry, 171, 395–397. 10.1176/appi.ajp.2014.14020138 24687194

[c63] Insel T., Cuthbert B., Garvey M., Heinssen R., Pine D. S., Quinn K., . . . Wang P. (2010). Research domain criteria (RDoC): Toward a new classification framework for research on mental disorders. The American Journal of Psychiatry, 167, 748–751. 10.1176/appi.ajp.2010.09091379 20595427

[c64] Jones E. B., & Sharpe L. (2017). Cognitive bias modification: A review of meta-analyses. Journal of Affective Disorders, 223, 175–183. 10.1016/j.jad.2017.07.034 28759865

[c65] *Käll A., Shafran R., Nygren T., Bennett S., Cooper Z., Coughtrey A. E., & Andersson G. (2020). A common elements approach to the development of a modular cognitive behavioural theory for chronic loneliness. Journal of Consulting and Clinical Psychology, 88, 269–282. 10.1037/ccp0000454 32068427

[c66] Kawa S., & Giordano J. (2012). A brief historicity of the *Diagnostic and Statistical Manual of Mental Disorders*: Issues and implications for the future of psychiatric canon and practice. Philosophy, Ethics, and Humanities in Medicine, 7, 2 10.1186/1747-5341-7-2 PMC3282636 22243976

[c67] Kendler K. S. (2008). Explanatory models for psychiatric illness. The American Journal of Psychiatry, 165, 695–702. 10.1176/appi.ajp.2008.07071061 18483135 PMC2744075

[c68] Kendler K. S. (2009). An historical framework for psychiatric nosology. Psychological Medicine, 39, 1935–1941. 10.1017/S0033291709005753 19368761 PMC2783473

[c69] Kendler K. S. (2012). The dappled nature of causes of psychiatric illness: Replacing the organic-functional/hardware-software dichotomy with empirically based pluralism. Molecular Psychiatry, 17, 377–388. 10.1038/mp.2011.182 22230881 PMC3312951

[c70] Kendler K. S., Aggen S. H., Knudsen G. P., Røysamb E., Neale M. C., & Reichborn-Kjennerud T. (2011). The structure of genetic and environmental risk factors for syndromal and subsyndromal common *DSM–IV* axis I and all axis II disorders. The American Journal of Psychiatry, 168, 29–39. 10.1176/appi.ajp.2010.10030340 20952461 PMC3126864

[c71] Kessler R. C., Chiu W. T., Demler O., Merikangas K. R., & Walters E. E. (2005). Prevalence, severity, and comorbidity of 12-month *DSM–IV* disorders in the National Comorbidity Survey Replication. Archives of General Psychiatry, 62, 617–627. 10.1001/archpsyc.62.6.617 15939839 PMC2847357

[c72] Keyes K. M., Eaton N. R., Krueger R. F., McLaughlin K. A., Wall M. M., Grant B. F., & Hasin D. S. (2012). Childhood maltreatment and the structure of common psychiatric disorders. The British Journal of Psychiatry, 200, 107–115. 10.1192/bjp.bp.111.093062 22157798 PMC3269653

[c73] Khoury B., Langer E. J., & Pagnini F. (2014). The *DSM*: Mindful science or mindless power? A critical review. Frontiers in Psychology, 5, 602 10.3389/fpsyg.2014.00602 24987385 PMC4060802

[c74] Kotov R., Krueger R. F., & Watson D. (2018). A paradigm shift in psychiatric classification: The Hierarchical Taxonomy Of Psychopathology (HiTOP). World Psychiatry, 17, 24–25. 10.1002/wps.20478 29352543 PMC5775140

[c75] Kotov R., Krueger R. F., Watson D., Achenbach T. M., Althoff R. R., Bagby R. M., . . . Zimmerman M. (2017). The Hierarchical Taxonomy of Psychopathology (HiTOP): A dimensional alternative to traditional nosologies. Journal of Abnormal Psychology, 126, 454–477. 10.1037/abn0000258 28333488

[c76] Kraemer H. C. (2007). *DSM* categories and dimensions in clinical and research contexts. International Journal of Methods in Psychiatric Research, 16, S8–S15. 10.1002/mpr.211 17623398 PMC6879071

[c77] Krueger R. F., Kotov R., Watson D., Forbes M. K., Eaton N. R., Ruggero C. J., . . . Zimmermann J. (2018). Progress in achieving quantitative classification of psychopathology. World Psychiatry, 17, 282–293. 10.1002/wps.20566 30229571 PMC6172695

[c78] Kupfer D. J., First M. B., & Regier D. E. (Eds.). (2002). A research agenda for DSM–V. Washington, DC: American Psychiatric Association.

[c79] Kuyken W., Watkins E., Holden E., White K., Taylor R. S., Byford S., . . . Dalgleish T. (2010). How does mindfulness-based cognitive therapy work? Behaviour Research and Therapy, 48, 1105–1112. 10.1016/j.brat.2010.08.003 20810101

[c80] Lahey B. B., Applegate B., Hakes J. K., Zald D. H., Hariri A. R., & Rathouz P. J. (2012). Is there a general factor of prevalent psychopathology during adulthood? Journal of Abnormal Psychology, 121, 971–977. 10.1037/a0028355 22845652 PMC4134439

[c81] Lehavot K., & Simoni J. M. (2011). The impact of minority stress on mental health and substance use among sexual minority women. Journal of Consulting and Clinical Psychology, 79, 159–170. 10.1037/a0022839 21341888 PMC4059829

[c82] Lorenzo-Luaces L., & DeRubeis R. J. (2018). Miles to go before we sleep: Advancing the understanding of psychotherapy by modelling complex processes. Cognitive Therapy and Research, 42, 212–217. 10.1007/s10608-018-9893-x

[c84] Luborsky L., Singer B., & Luborsky L. (1975). Comparative studies of psychotherapies. Is it true that “everywon has one and all must have prizes”? Archives of General Psychiatry, 32, 995–1008. 10.1001/archpsyc.1975.01760260059004 239666

[c85] MacLeod C., & Clarke P. J. F. (2015). The attentional bias modification approach to anxiety intervention. Clinical Psychological Science, 3, 58–78. 10.1177/2167702614560749

[c86] MacLeod C., Rutherford E., Campbell L., Ebsworthy G., & Holker L. (2002). Selective attention and emotional vulnerability: Assessing the causal basis of their association through the experimental manipulation of attentional bias. Journal of Abnormal Psychology, 111, 107–123. 10.1037/0021-843X.111.1.107 11866165

[c87] Maj M. (2005). “Psychiatric comorbidity”: An artefact of current diagnostic systems? The British Journal of Psychiatry, 186, 182–184. 10.1192/bjp.186.3.182 15738496

[c88] Mansell W. (2019). Transdiagnostic psychiatry goes above and beyond classification. World Psychiatry, 18, 360–361. 10.1002/wps.20680 31496093 PMC6732685

[c89] Marecek J., & Hare-Mustin R. T. (2009). Clinical psychology: The politics of madness In Fox D., Prilleltensky I., & Austin S. (Eds.), Critical psychology: An introduction (pp. 75–92). Thousand Oaks, CA: Sage Ltd.

[c90] Markon K. E., Chmielewski M., & Miller C. J. (2011). The reliability and validity of discrete and continuous measures of psychopathology: A quantitative review. Psychological Bulletin, 137, 856–879. 10.1037/a0023678 21574681

[c91] Masyn K. E., Henderson C. E., & Greenbaum P. E. (2018). Exploring the latent structures of psychological constructs in social development using the dimensional-categorical spectrum. Social Development, 12, 82–86. 10.1111/j.1467-9507.2009.00573.x PMC3905984 24489441

[c92] McEvoy P. M., Nathan P., & Norton P. J. (2009). Efficacy of transdiagnostic treatments: A review of published outcome studies and future research directions. Journal of Cognitive Psychotherapy, 23, 20–33. 10.1891/0889-8391.23.1.20

[c93] McNally R. J. (2016). Can network analysis transform psychopathology? Behaviour Research and Therapy, 86, 95–104. 10.1016/j.brat.2016.06.006 27424882

[c94] McTeague L. M., Huemer J., Carreon D. M., Jiang Y., Eickhoff S. B., & Etkin A. (2017). Identification of common neural circuit disruptions in cognitive control across psychiatric disorders. The American Journal of Psychiatry, 174, 676–685. 10.1176/appi.ajp.2017.16040400 28320224 PMC5543416

[c95] Meidlinger P. C., & Hope D. A. (2017). The new transdiagnostic cognitive behavioral treatments: Commentary for clinicians and clinical researchers. Journal of Anxiety Disorders, 46, 101–109. 10.1016/j.janxdis.2016.11.002 27856069

[c96] Meyer A. (2017). A biomarker of anxiety in children and adolescents: A review focusing on the error-related negativity (ERN) and anxiety across development. Developmental Cognitive Neuroscience, 27, 58–68. 10.1016/j.dcn.2017.08.001 28818707 PMC6987910

[c148] Mogg K., Mathews A., & Eysenck M. (1992). Attentional bias to threat in clinical anxiety states. Cognition and Emotion, 6, 149–159. 10.1080/02699939208411064

[c97] Morris S. E., & Cuthbert B. N. (2012). Research domain criteria: Cognitive systems, neural circuits, and dimensions of behavior. Dialogues in Clinical Neuroscience, 14, 29–37. 22577302 10.31887/DCNS.2012.14.1/smorris PMC3341647

[c98] Moses E. B., & Barlow D. H. (2006). A new unified treatment approach for emotional disorders based on emotion science. Current Directions in Psychological Science, 15, 146–150. 10.1111/j.0963-7214.2006.00425.x

[c99] National Collaborating Centre for Mental Health (U. K.) (2011). Common mental health disorders: Identification and pathways to care (NICE Clinical Guidelines, No. 123). Leicester, UK: British Psychological Society Retrieved from https://www.ncbi.nlm.nih.gov/books/NBK92254/22536621

[c100] Newby J. M., McKinnon A., Kuyken W., Gilbody S., & Dalgleish T. (2015). Systematic review and meta-analysis of transdiagnostic psychological treatments for anxiety and depressive disorders in adulthood. Clinical Psychology Review, 40, 91–110. 10.1016/j.cpr.2015.06.002 26094079

[c101] Newby J. M., Twomey C., Yuan Li S. S., & Andrews G. (2016). Transdiagnostic computerised cognitive behavioural therapy for depression and anxiety: A systematic review and meta-analysis. Journal of Affective Disorders, 199, 30–41. 10.1016/j.jad.2016.03.018 27060430

[c149] Norcross J. C., & Wampold B. E. (2011). Evidence-based therapy relationships: Research conclusions and clinical practices. Psychotherapy, 48, 98–102. 10.1037/a0022161 21401280

[c102] Norton P. J., Hayes S. A., & Hope D. A. (2004). Effects of a transdiagnostic group treatment for anxiety on secondary depression. Depression and Anxiety, 20, 198–202. 10.1002/da.20045 15643648

[c103] Oquendo M. A., Galfalvy H., Russo S., Ellis S. P., Grunebaum M. F., Burke A., & Mann J. J. (2004). Prospective study of clinical predictors of suicidal acts after a major depressive episode in patients with major depressive disorder or bipolar disorder. The American Journal of Psychiatry, 161, 1433–1441. 10.1176/appi.ajp.161.8.1433 15285970

[c104] Otowa T., Hek K., Lee M., Byrne E. M., Mirza S. S., Nivard M. G., . . . Hettema J. M. (2016). Meta-analysis of genome-wide association studies of anxiety disorders. Molecular Psychiatry, 21, 1391–1399. 10.1038/mp.2015.197 26754954 PMC4940340

[c105] Păsărelu C. R., Andersson G., Bergman Nordgren L., & Dobrean A. (2017). Internet-delivered transdiagnostic and tailored cognitive behavioral therapy for anxiety and depression: A systematic review and meta-analysis of randomized controlled trials. Cognitive Behaviour Therapy, 46, 1–28. 10.1080/16506073.2016.1231219 27712544

[c106] Pearl S. B., & Norton P. J. (2017). Transdiagnostic versus diagnosis specific cognitive behavioural therapies for anxiety: A meta-analysis. Journal of Anxiety Disorders, 46, 11–24. 10.1016/j.janxdis.2016.07.004 27466074

[c107] Pettersson E., Larsson H., & Lichtenstein P. (2016). Common psychiatric disorders share the same genetic origin: A multivariate sibling study of the Swedish population. Molecular Psychiatry, 21, 717–721. 10.1038/mp.2015.116 26303662

[c108] Pilling S., Whittington C., Taylor C., & Kendrick T. (2011). Identification and care pathways for common mental health disorders: Summary of NICE guidance. British Medical Journal, 342, d2868 10.1136/bmj.d2868 21610049

[c109] Power M. J., & Dalgleish T. (2015). Cognition and emotion: From order to disorder. London, UK: Psychology Press 10.4324/9781315708744

[c110] Proctor E. K., Landsverk J., Aarons G., Chambers D., Glisson C., & Mittman B. (2009). Implementation research in mental health services: An emerging science with conceptual, methodological, and training challenges. Administration and Policy in Mental Health, 36, 24–34. 10.1007/s10488-008-0197-4 19104929 PMC3808121

[c111] Rapaport M. H., Clary C., Fayyad R., & Endicott J. (2005). Quality-of-life impairment in depressive and anxiety disorders. The American Journal of Psychiatry, 162, 1171–1178. 10.1176/appi.ajp.162.6.1171 15930066

[c112] Regier D. A., Kuhl E. A., & Kupfer D. J. (2013). The *DSM–5*: Classification and criteria changes. World Psychiatry, 12, 92–98. 10.1002/wps.20050 23737408 PMC3683251

[c113] Regier D. A., Narrow W. E., Clarke D. E., Kraemer H. C., Kuramoto S. J., Kuhl E. A., & Kupfer D. J. (2013). *DSM–5* field trials in the United States and Canada, Part II: Test-retest reliability of selected categorical diagnoses. The American Journal of Psychiatry, 170, 59–70. 10.1176/appi.ajp.2012.12070999 23111466

[c114] Reinholt N., & Krogh J. (2014). Efficacy of transdiagnostic cognitive behaviour therapy for anxiety disorders: A systematic review and meta-analysis of published outcome studies. Cognitive Behaviour Therapy, 43, 171–184. 10.1080/16506073.2014.897367 24646219

[c115] Ruggero C. J., Kotov R., Hopwood C. J., First M., Clark L. A., Skodol A. E., . . . Zimmermann J. (2019). Integrating the Hierarchical Taxonomy of Psychopathology (HiTOP) into clinical practice. Journal of Consulting and Clinical Psychology, 87, 1069–1084. 10.1037/ccp0000452 31724426 PMC6859953

[c116] Santor D. A., Gregus M., & Welch A. (2006). FOCUS ARTICLE: Eight Decades of Measurement in Depression. Measurement: Interdisciplinary Research and Perspectives, 4, 135–155. 10.1207/s15366359mea0403_1

[c117] Sauer-Zavala S., Gutner C. A., Farchione T. J., Boettcher H. T., Bullis J. R., & Barlow D. H. (2017). Current definitions of “transdiagnostic” in treatment development: A search for consensus. Behavior Therapy, 48, 128–138. 10.1016/j.beth.2016.09.004 28077216

[c118] *Schweizer T. H., Snyder H. R., Young J. F., & Hankin B. L. (2020). The breadth and potency of transdiagnostic cognitive risks for psychopathology in youth. Journal of Consulting and Clinical Psychology, 88, 196–211. 10.1037/ccp0000470 PMC7219569 32068422

[c119] Shanmugan S., Wolf D. H., Calkins M. E., Moore T. M., Ruparel K., Hopson R. D., . . . Satterthwaite T. D. (2016). Common and dissociable mechanisms of executive system dysfunction across psychiatric disorders in youth. The American Journal of Psychiatry, 173, 517–526. 10.1176/appi.ajp.2015.15060725 26806874 PMC4886342

[c120] Sharma A., Wolf D. H., Ciric R., Kable J. W., Moore T. M., Vandekar S. N., . . . Satterthwaite T. D. (2017). Common dimensional reward deficits across mood and psychotic disorders: A connectome-wide association study. The American Journal of Psychiatry, 174, 657–666. 10.1176/appi.ajp.2016.16070774 28135847 PMC5495611

[c121] Slade T., & Watson D. (2006). The structure of common *DSM–IV* and ICD-10 mental disorders in the Australian general population. Psychological Medicine, 36, 1593–1600. 10.1017/S0033291706008452 16882356

[c122] Snyder H. R., Young J. F., & Hankin B. L. (2017). Strong homotypic continuity in common psychopathology, internalizing and externalizing specific factors over time in adolescents. Clinical Psychological Science, 5, 98–110. 10.1177/2167702616651076 28239532 PMC5320894

[c123] Steele S. J., Farchione T. J., Cassiello-Robbins C., Ametaj A., Sbi S., Sauer-Zavala S., & Barlow D. H. (2018). Efficacy of the Unified Protocol for transdiagnostic treatment of comorbid psychopathology accompanying emotional disorders compared to treatments targeting single disorders. Journal of Psychiatric Research, 104, 211–216. 10.1016/j.jpsychires.2018.08.005 30103069 PMC6219859

[c124] Sunderland M., Carragher N., Chapman C., Mills K., Teesson M., Lockwood E., . . . Slade T. (2016). The shared and specific relationships between exposure to potentially traumatic events and transdiagnostic dimensions of psychopathology. Journal of Anxiety Disorders, 38, 102–109. 10.1016/j.janxdis.2016.02.001 26874292

[c125] Swartz J. R., Knodt A. R., Radtke S. R., & Hariri A. R. (2015). A neural biomarker of psychological vulnerability to future life stress. Neuron, 85, 505–511. 10.1016/j.neuron.2014.12.055 25654256 PMC4319095

[c126] Teasdale J. D. (1988). Cognitive vulnerability to persistent depression. Cognition and Emotion, 2, 247–274. 10.1080/02699938808410927

[c127] Ussher J. M. (2010). Are we medicalizing women’s misery? A critical review of women’s higher rates of reported depression. Feminism & Psychology, 20, 9–35. 10.1177/0959353509350213

[c128] van Loo H. M., & Romeijn J. W. (2015). Psychiatric comorbidity: Fact or artifact? Theoretical Medicine and Bioethics, 36, 41–60. 10.1007/s11017-015-9321-0 25636962 PMC4320768

[c129] Wahl K., Ehring T., Kley H., Lieb R., Meyer A., Kordon A., . . . Schönfeld S. (2019). Is repetitive negative thinking a transdiagnostic process? A comparison of key processes of RNT in depression, generalized anxiety disorder, obsessive-compulsive disorder, and community controls. Journal of Behavior Therapy and Experimental Psychiatry, 64, 45–53. 10.1016/j.jbtep.2019.02.006 30851652

[c130] Wakefield J. C. (2014). Wittgenstein’s nightmare: Why the RDoC grid needs a conceptual dimension. World Psychiatry, 13, 38–40. 10.1002/wps.20097 24497242 PMC3918013

[c131] Waszczuk M. A., Zimmerman M., Ruggero C., Li K., MacNamara A., Weinberg A., . . . Kotov R. (2017). What do clinicians treat: Diagnoses or symptoms? The incremental validity of a symptom-based, dimensional characterization of emotional disorders in predicting medication prescription patterns. Comprehensive Psychiatry, 79, 80–88. 10.1016/j.comppsych.2017.04.004 28495012 PMC5643213

[c132] Watkins E. R. (2016). Rumination-focused cognitive-behavioural therapy for depression. New York, NY: Guilford Press.

[c133] Watkins E. (2018). Examining transdiagnostic interventions: Reviewing conceptual and methodological issues. Keynote address delivered at the 2018 Conference on Transdiagnostic Approaches to Mental Health Challenges, Cambridge, UK.

[c134] Weinberg A., Kotov R., & Proudfit G. H. (2015). Neural indicators of error processing in generalized anxiety disorder, obsessive-compulsive disorder, and major depressive disorder. Journal of Abnormal Psychology, 124, 172–185. 10.1037/abn0000019 25384068

[c135] Wells A., & Matthews G. (1994). Attention and emotion: A clinical perspective. Hillsdale, NJ: Erlbaum.

[c136] Werner-Seidler A., Hitchcock C., Bevan A., McKinnon A., Gillard J., Dahm T., . . . Dalgleish T. (2018). A cluster randomized controlled platform trial comparing group MEmory specificity training (MEST) to group psychoeducation and supportive counselling (PSC) in the treatment of recurrent depression. Behaviour Research and Therapy, 105, 1–9. 10.1016/j.brat.2018.03.004 29587159 PMC5937852

[c137] Widiger T. A., & Samuel D. B. (2005). Diagnostic categories or dimensions? A question for the *diagnostic and statistical manual of mental disorders*–5th edition. Journal of Abnormal Psychology, 114, 494–504. 16351373 10.1037/0021-843X.114.4.494

[c138] Wiggins J. L., Mitchell C., Hyde L. W., & Monk C. S. (2015). Identifying early pathways of risk and resilience: The codevelopment of internalizing and externalizing symptoms and the role of harsh parenting. Development and Psychopathology, 27, 1295–1312. 10.1017/S0954579414001412 26439075 PMC4961476

[c139] Wittchen H. U., Carter R. M., Pfister H., Montgomery S. A., & Kessler R. C. (2000). Disabilities and quality of life in pure and comorbid generalized anxiety disorder and major depression in a national survey. International Clinical Psychopharmacology, 15, 319–328. 10.1097/00004850-200015060-00002 11110007

[c140] Wittchen H. U., Jacobi F., Rehm J., Gustavsson A., Svensson M., Jönsson B., . . . Steinhausen H. C. (2011). The size and burden of mental disorders and other disorders of the brain in Europe 2010. European Neuropsychopharmacology, 21, 655–679. 10.1016/j.euroneuro.2011.07.018 21896369

[c141] Wittchen H. U., Nocon A., Beesdo K., Pine D. S., Hofler M., Lieb R., & Gloster A. T. (2008). Agoraphobia and panic. Prospective-longitudinal relations suggest a rethinking of diagnostic concepts. Psychotherapy and Psychosomatics, 77, 147–157. 10.1159/000116608 18277061

[c142] *Woods W. C., Arizmendi C., Gates K. M., Stepp S. D., Pilkonis P. A., & Wright A. (2020). Personalized models of psychopathology as contextualized dynamic processes: An example from individuals with borderline personality disorder. Journal of Consulting and Clinical Psychology, 88, 240–254. 10.1037/ccp0000472 PMC7034576 32068425

