# Supplementary material for: Transdiagnostic Approaches to Mental Health Problems: Current Status and Future Directions
Source: J Consult Clin Psychol. 2020 Mar;88(3):179–95. doi: 10.1037/ccp0000482 (PMC7027356; doi:10.1037/ccp0000482)
Supplement: Supplementary file 1 [file Transdiagnostic.docx]

SUPPLEMENTARY MATERIALS

Transdiagnostic approaches to mental health problems: Current status and future directions.

Tim Dalgleish+

Melissa Black+

David Johnston

Anna Bevan

Medical Research Council Cognition and Brain Sciences, University of Cambridge

and the Cambridgeshire and Peterborough NHS Foundation Trust

+ To be considered as joint first authors

**Table S1. Research Domain Criteria (RDoC) Matrix – Domains, Constructs and Sub-constructs (Insel et al., 2010)**

| **SYSTEM** | **Construct/Subconstruct** |
| --- | --- |
| **Negative Valence Systems** | - Acute Threat (“Fear”) - Potential Threat (“Anxiety”) - Sustained Threat - Loss - Frustrative Non-Reward |
| **Positive Valence Systems** | - Reward Responsiveness - Reward Anticipation - Initial Response to Reward - Reward Satiation - Reward Learning - Probabilistic and Reinforcement Learning - Reward Prediction Error - Habit (Positive Valence System) - Reward Valuation - Reward (probability) - Delay - Effort |
| **Cognitive Systems** | - Attention - Perception - Visual perception - Auditory perception - Olfactory/Somatosensory/Multimodal Perception - Declarative memory - Language - Cognitive Control - Goal Selection; Updating, Representation, and Maintenance - Response Selection; Inhibition/Suppression - Performance Monitoring - Working Memory - Active Maintenance - Flexible Updating - Limited Capacity - Interference Control |
| **Systems for Social Processes** | - Affiliation and attachment - Social communication - Reception of Facial Communication - Production of Facial Communication - Reception of Non-Facial Communication - Production of Non-Facial Communication - Perception and Understanding of Self - Agency - Self-Knowledge - Perception and Understanding of Others - Animacy Perception - Action Perception - Understanding Mental States |
| **Arousal and Regulatory Systems** | - Arousal - Circadian Rhythms - Sleep-Wakefulness |
| **Sensorimotor Systems** | - Motor Actions - Action, Planning and Selection - Sensorimotor Dynamics - Initiation - Execution - Inhibition and Termination - Agency and Ownership - Habit - Sensorimotor - Innate Motor Patterns |

**Table S2. Transdiagnostic Processes identified by Harvey et al. (2004)**

| **Attention** | **Memory** | **Reasoning** | **Thought** | **Behaviour** |
| --- | --- | --- | --- | --- |
| Selective attention (external) | Explicit selective memory | Interpretational bias | Recurrent negative thinking | Avoidance |
| Selective attention (internal) | Recurrent memories | Expectancy bias | Positive and negative metacognitive beliefs | Safety behaviours |
| Attentional avoidance | Overgeneral memory | Emotional reasoning | Thought suppression |  |

**Table S3. Components of Existing Transdiagnostic Interventions**

| **Unified Protocol (Barlow et al., 2010)** | **MATCH Protocol (Chorpita & Weisz, 2009)** | **Shaping Healthy Minds (Black et al., 2018)** | **Process-based CBT (Hayes & Hofmann, 2018)** |
| --- | --- | --- | --- |
| **Treatment Modules** | **Treatment Modules** | **Treatment Modules** | **Treatment processes** |
| Module 1: Psychoeducation and Treatment Rationale | CBT Essentials | Module 1:  Getting Acquainted with Shaping Healthy Minds | Contingency Management |
| Module 2: Motivational Enhancement | Getting Acquainted – Anxiety | Module 2:  Understanding Emotions | Stimulus Control |
| Module 3:  Emotional Awareness | Fear Ladder | Module 3:  Managing and tolerating emotions | Shaping |
| Module 4:  Cognitive Appraisal and Reappraisal | Learning about Anxiety – Child | Module 4: Behavioural Activation | Self-management |
| Module 5: Countering Emotion-Driven behaviours and Emotional Avoidance | Learning about Anxiety – Parent | Module 5:  Overcoming Avoidance | Arousal reduction |
| Module 6: Interoceptive and Situational Exposure | Practicing | Module 6:  Tackling Unhelpful Thoughts | Coping and Emotion Regulation |
| Module 7: Conclusion and Relapse Prevention | Maintenance | Module 7:  Tackling Unhelpful Habits | Problem-solving |
|  | Cognitive – STOP | Module 8:  Overcoming Repetitive Thinking | Exposure Strategies |
|  | Wrap Up | Module 9:  Managing upsetting memories and images | Behavioural Activation |
|  | Getting Acquainted – Depression | Module 10:  Relapse Prevention and Future Orientation | Interpersonal Skills |
|  | Learning about Depression – Child |  | Cognitive Reappraisal |
|  | Learning about Depression – Parent |  | Modifying Core Beliefs |
|  | Problem Solving |  | Cognitive Defusion |
|  | Activity Selection |  | Cultivating Psychological Acceptance |
|  | Learning to Relax |  | Values Choice and Clarification |
|  | Quick Calming |  | Mindfulness Practice |
|  | Presenting a Positive Self |  | Enhancing Motivation |
|  | Cognitive Coping – BLUE |  | Crisis Management and Treating Suicidality from a Behavioural Perspective |
|  | Cognitive Coping – TLC |  |  |
|  | Plans for Coping |  |  |
|  | Safety Planning |  |  |
|  | Trauma Narrative |  |  |
|  | Engaging Parents |  |  |
|  | Learning about Behaviour |  |  |
|  | One-on-One Time |  |  |
|  | Praise |  |  |
|  | Active Ignoring |  |  |
|  | Giving Effective Instructions |  |  |
|  | Rewards |  |  |
|  | Time Out |  |  |
|  | Making a Plan |  |  |
|  | Daily Report Card |  |  |
|  | Looking Ahead |  |  |
|  | Booster Session |  |  |

**Table 4. Reviews of transdiagnostic treatment research**

| **Review** | **Target Interventions** | **Target populations** | **No. of studies** | **Key findings** | **Support for Transdiagnostic Approach?** | **Average Effects?** |
| --- | --- | --- | --- | --- | --- | --- |
| Anderson, Bland, Toner, & McMillan (2016) | Transdiagnostic CBT for Depression and Anxiety | Adults with Depression and Anxiety | 8 (Systematic Review)  4 (Meta-analysis) | Transdiagnostic CBT > Control;  Transdiagnostic CBT = Active Treatment. | Positive signs for value of transdiagnostic CBT, but insufficient evidence to warrant replacing disorder-specific CBT.  Noted that studies comparing effectiveness of transdiagnostic and disorder-specific CBT were needed. | SMD for Transdiagnostic CBT vs. Control (N = 4):  Anxiety, SMD = -0.90  Depression, SMD = -0.64  Generic (DASS-21; N = 3) = -0.77  (effect sizes could not be calculated as most studies gave treatment to waitlist following trial period) |
| Newby, McKinnon, Kuyken, Gilbody, & Dalgleish (2015) | Transdiagnostic Psychological Treatments for Depressive and Anxiety Disorders | Adults with depressive and anxiety disorders | 50 | Transdiagnostic CBT > Control;  Transdiagnostic treatments are efficacious; high heterogeneity amongst treatment effects.  Preliminary results (N = 4) suggested Transdiagnostic CBT > Disorder-specific CBT. | Transdiagnostic treatments are efficacious (large overall mean uncontrolled effects for anxiety and depression).  Noted that higher quality studies are needed to explore treatment effects. | Uncontrolled ES for pre- to post-treatment:  Anxiety *g* = .85  Depression *g* = .91  Quality of Life *g* = .69  Controlled ES for RCTs (N = 24):  Anxiety *g* = .65  Depression *g* = .80  Quality of Life *g* = .46 |
| Newby, Twomey, Li, & Andrews (2016) | Transdiagnostic computerised CBT for depression and anxiety | Adults with depression and anxiety | 17 | Transdiagnostic cCBT > Control  Preliminary results (N = 4) suggested that Transdiagnostic cCBT > Disorder-specific cCBT for depression and Quality of Life; Transdiagnostic cCBT = Disorder-specific cCBT for Anxiety. | Transdiagnostic computerised CBT is efficacious, comparable to disorder-specific cCBT. | Controlled ES for RCTs (N = 17):  Anxiety *g* = .84  Depression *g* = .78  Quality of Life *g* = .48 |
| Pasarelu, Andersson, Bergman Nordgren, & Dobrean (2016) | Internet-delivered transdiagnostic and tailored CBT for anxiety and depression | Adults with Anxiety and Depression | 19 | Transdiagnostic and Individually-tailored CBT are effective  Transdiagnostic/Tailored CBT = Disorder-specific CBT for Anxiety  Transdiagnostic/Tailored CBT > Disorder-specific CBT for depression and quality of life | Transdiagnostic interventions are effective for anxiety disorders and depression; Some evidence transdiagnostic treatment was more effective at treating depression symptoms. | Overall sample of studies pre-post outcomes:  Anxiety *g* = 1.06 (95% CI: .91–1.22)  Depression *g* = 1.08 (95% CI: .88–1.28)  Quality of life *g* = .63 (95% CI: .53–.73)  Transdiagnostic/tailored iCBT compared with control:  Anxiety *g* = .82, 95% CI: .58–1.05  Depression *g*  =  .79, 95% CI: .59–1.00  Quality of Life: *g* = .55, 95% CI: .37–.73 |
| Pearl & Norton (2016) | Transdiagnostic vs. Diagnosis-specific CBT for anxiety | Adults with anxiety | 80 | Transdiagnostic CBT = Disorder-specific CBT | Results suggest equivalence of transdiagnostic and diagnosis-specific CBT outcomes. | Overall sample of studies:  *g* = 0.966 (95% CI: 0.896–1.037).  tCBT, *g* = 1.059 (95% CI: 0.876–1.242)  dxCBT, *g* = 0.951 (95% CI: 0.874–1.027) |
| Reinholt & Krogh (2014) | Transdiagnostic CBT for Anxiety Disorders | Adults with Anxiety Disorders | 12 | Transdiagnostic CBT > Wait-list and treatment-as-usual.  Transdiagnostic CBT = Disorder-specific CBT  Treatment gains were maintained through follow-up. | Cautious supportive evidence for the efﬁcacy of Transdiagnostic CBT for anxiety disorders.  Large heterogeneity suggesting differences in treatment effects between the studies. | SMD for Transdiagnostic CBT vs. Control (N = 12):  Anxiety, SMD = -0.68  SMD for Transdiagnostic CBT vs. Control for RCTs (N = 6):  Anxiety, SMD = -0.79  SMD for Transdiagnostic CBT vs. Control for Observational and Controlled studies (N = 6):  Anxiety, SMD = -0.67 |

Note. A systematic review was conducted by Fusar-Poli et al. (2019), focusing on any original articles that included the word “transdiagnostic” in the title. We have not included the review in this table due to its focus on titles only with no systematic inclusion criteria and the heterogeneity of included studies which limited meaningful comparisons or conclusions.

References for Supplemental Material

Andersen, P., Toner, P., Bland, M., & McMillan, D. (2016). Effectiveness of Transdiagnostic Cognitive Behaviour Therapy for Anxiety and Depression in Adults: A Systematic Review and Meta-analysis. *Behavioral and Cognitive Psychotherapy, 44(6),* 673–90. doi: 10.1017/S1352465816000229

Barlow, D.H., Ellard, K.K., Fairholme, C.P., Farchione, T.J., Boisseau, C.L., Allan, L.B., Ehrenreich-May, J.T. (2010). Unified Protocol for Transdiagnostic Treatment of Emotional Disorders: Workbook (First Edition). Oxford University Press: Oxford, UK. doi: 10.1093/med:psych/9780199772674.001.0001

Black, M.J., Hitchcock, C., Bevan, A., O Leary, C., Clarke, J., Elliott, R., Watson, P., LaFortune, L., Rae, S., Gilbody, S., Kuyken, W., Johnston, D., Newby, J., & Dalgleish, T. (2018). The HARMONIC trial: study protocol for a randomised controlled feasibility trial of Shaping Healthy Minds—a modular transdiagnostic intervention for mood, stressor-related and anxiety disorders in adults. *BMJ Open, 8,* e024546. doi: 10.1136/bmjopen-2018-024546

Chorpita, B.F. & Weisz, J.R. (2009). Modular Approach to Therapy for Children with Anxiety, Depression, Trauma, or Conduct Problems (MATCH-ADTC). Satellite Beach, FL: PracticeWise, LLC.

Harvey, A.G., Watkins, E., Mansell, W., & Shafran, R. eds. (2004). Cognitive behavioural processes across psychological disorders: A transdiagnostic approach to research and treatment. Oxford University Press: Oxford.

Hayes, S. C. & Hofmann, S. G. (Eds.) (2018). Process-based CBT: The science and core clinical competencies of cognitive behavioral therapy. Oakland, CA: New Harbinger Publications. ISBN-13: 978-1626255968.

Insel, T., Cuthbert, B., Garvey, M., Heinssen, R., Pine, D.S., Quinn, K., Sanislow, C., & Wang, P. (2010). Research domain criteria (RDoC): toward a new classification framework for research on mental disorders. *American Journal of Psychiatry, 167*, 748-51. doi: 10.1176/appi.ajp.2010.09091379.

Newby, J.M., McKinnon, A., Kuyken, W., Gilbody, S., & Dalgleish, T. (2015). Systematic review and meta-analysis of transdiagnostic psychological treatments for anxiety and depressive disorders in adulthood. *Clinical Psychology Review, 40,* 91–110. doi: 10.1016/j.cpr.2015.06.002

Newby, J.M., Twomey, C., Li, S., & Andrews, G. (2016). Transdiagnostic computerised cognitive behavioural therapy for depression and anxiety: A systematic review and meta-analysis. *Journal of Affective Disorders, 199,* 30–41. doi: 10.1016/j.jad.2016.03.018

Păsărelu, C.R., Andersson, G., Bergman Nordgren, L., & Dobrean, A. (2016). Internet-delivered transdiagnostic and tailored cognitive behavioral therapy for anxiety and depression: a systematic review and meta-analysis of randomized controlled trials. *Cognitive Behaviour Therapy, 46(1)*, 1–28. doi: 10.1080/16506073.2016.1231219

Pearl, S.B., & Norton, P.J. (2016). Transdiagnostic versus diagnosis specific cognitive behavioural therapies for anxiety: A meta-analysis. *Journal of Anxiety Disorders, 46*, 11–24. doi: 10.1016/j.janxdis.2016.07.004

Reinholt, N., & Krogh, J. (2014). Efficacy of Transdiagnostic Cognitive Behaviour Therapy for Anxiety Disorders: A Systematic Review and Meta-Analysis of Published Outcome Studies. *Cognitive Behaviour Therapy, 43(3)*:171–84. doi: 10.1080/16506073.2014.897367
